# Phosphoregulation of interleukin-1 receptor-associated kinase 1 in inflammatory signaling

**DOI:** 10.3389/fimmu.2026.1937314

**Published:** 2026-09-03

**Authors:** Manasa Suresh, Chrysilla Espy Vaz, Leona Dcunha, Rajesh Raju, Sneha M. Pinto, Saptami Kanekar

**Affiliations:** 1 Centre for Integrative Omics Data Science, Yenepoya (Deemed to be University), Mangalore, Karnataka, India; 2 School of Biosciences, Faculty of Health and Medical Sciences, University of Surrey, Guildford, United Kingdom

**Keywords:** cancer, inflammation, IRAK1, phosphoproteomics, phosphoregulation, post-translational modification

## Abstract

Interleukin-1 receptor-associated kinase 1 (IRAK1) is a serine/threonine kinase that functions as a central transducer of Toll-like receptor (TLR) and interleukin-1 receptor (IL-1R) signaling, linking pathogen recognition to the activation of nuclear factor kappa B (NF-κB), mitogen-activated protein kinases (MAPK), and interferon regulatory factors (IRFs). Although the role of IRAK1 in innate immune signaling is well established, its regulatory complexity remains incompletely characterized, particularly regarding the dynamic phosphorylation landscape that governs activation, substrate specificity, and signal termination. This review presents a systematic phosphoproteomics map of IRAK1 phosphorylation, integrating 213 profiling and 33 differential mass spectrometry-based phosphoproteomic datasets from 56 studies. Eighteen phosphosites were identified in the profiling datasets and 16 in the differential datasets across independent expression and acquisition conditions. Among these, Ser131 (detected in 128 profiling and 26 differential datasets) and Ser110 (detected in 26 profiling and nine differential datasets) were the most frequently observed sites. Most high-frequency phosphosites are located within the serine/threonine kinase domain and the C-terminal proline/serine/threonine-rich (ProST) regulatory region of the protein. However, frequent detection does not necessarily indicate functional significance, and these sites are potential candidates for targeted validation. In addition to canonical phosphorylation, this review evaluates the coordinated interplay among phosphorylation, K63/K48-linked ubiquitination, and SUMOylation, which together determine whether signaling is propagated or terminated. We also discuss the non-canonical functions of IRAK1 in transcriptional regulation, antiviral defense, cytoskeletal organization, and oncogenic signaling, and evaluate current pharmacological inhibitors of IRAK1, including Pacritinib, JH-X-119-01, 1, 4-naphthoquinone, and rosoxacin. Overall, this review provides a systematic, data-informed framework for prioritizing phosphosite-specific functional studies to identify phospho-dependent interaction interfaces as potential targets for precision clinical interventions and biomarker development in inflammatory diseases and cancer.

## 1 Introduction

**Figure 1:**
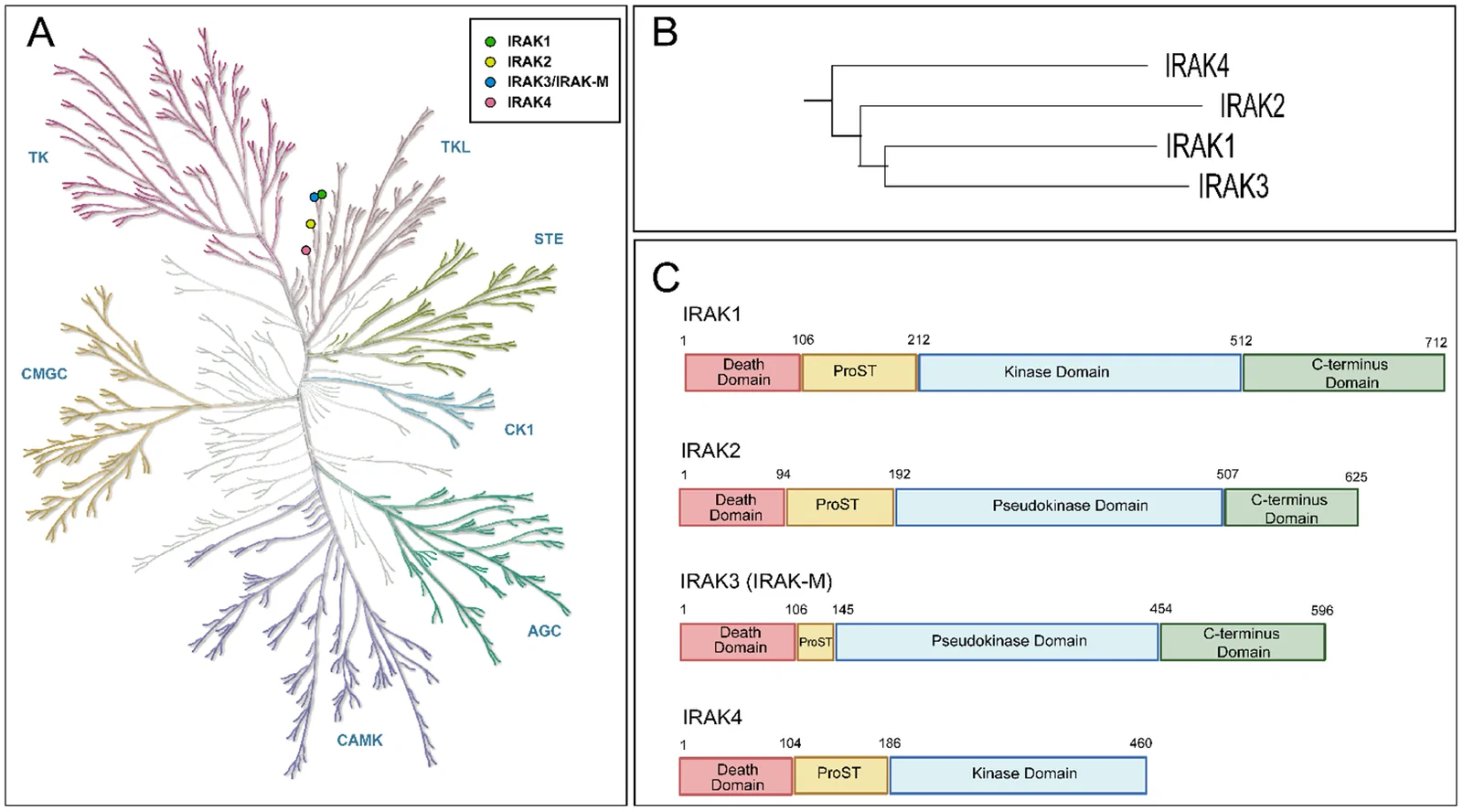
Phylogenetic and structural relationships among human IRAK family members. **(A)** Position of IRAK family kinases within the human kinome tree. IRAK1 (green), IRAK2 (yellow), IRAK3/IRAK-M (blue), and IRAK4 (pink) clustered within the tyrosine kinase-like (TKL) group. **(B)** Phylogenetic relationships among the four human IRAK paralogs. **(C)** Domain architecture of IRAK1, IRAK2, IRAK3 (IRAK-M), and IRAK4, drawn to scale according to amino acid position. Created in BioRender; Source: https://BioRender.com/c7n1ndx. All four proteins share an N-terminal death domain and a proline/serine/threonine-rich (ProST) linker region, followed by a central kinase or pseudokinase domain. IRAK1, IRAK2, and IRAK3 also retain a C-terminal domain required for TRAF6 recruitment and activation, which is absent in IRAK4. The kinome tree was generated using KinMap (http://www.kinhub.org/kinmap/).

The innate immune system depends on a precisely coordinated network of pattern recognition receptor (PRR) signaling cascades to initiate host defense against pathogens and endogenous danger signals. At the core of Toll-like receptor (TLR) and interleukin-1 family receptor (IL-1R) signal transduction lies the interleukin-1 receptor-associated kinase (IRAK) family, a group of four serine/threonine kinases, which comprises of IRAK1, IRAK2, IRAK3 (also known as IRAK-M), and IRAK4 (1–5). Although these proteins share a conserved N-terminal death domain (DD) and C-terminal kinase-like domain, they differ substantially in their catalytic competence. IRAK1 and IRAK4 are catalytically active serine/threonine kinases that mediate phosphorylation-dependent signal propagation downstream of TLR and IL-1R activation (6). In contrast, IRAK2 and IRAK3/IRAK-M contain divergent kinase-like domains with substitutions in residues that contribute to canonical kinase activity. IRAK2 was initially classified as a pseudokinase because of its low intrinsic kinase activity and replacement of the conserved catalytic residue, aspartate. However, subsequent studies have demonstrated that IRAK2 retains its kinase activity and can be activated by IRAK4-dependent phosphorylation. Therefore, IRAK2 is more accurately described as an atypical or kinase-impaired kinase rather than a strictly inactive pseudokinase (7). IRAK3/IRAK-M is generally considered a catalytically inactive pseudokinase that primarily functions as a regulator of IRAK-containing signaling complexes. Structural analysis of the IRAK3 pseudokinase domain revealed a closed, pseudoactive conformation and a distinctive dimeric arrangement, supporting the concept that IRAK3 mediates signaling through protein-protein interactions, allosteric regulation, and complex assembly rather than through typical canonical phosphotransferase activity (8). It comprises two catalytically active kinases (IRAK1 and IRAK4), one atypical kinase (IRAK2), and one catalytically inactive pseudokinase (IRAK3/IRAK-M) (1, 9–11) (**Figures 1A, B**). Despite these catalytic differences, all four proteins contribute to the organization and regulation of TLR and IL-1R signaling complexes. IRAK1 (also designated IRAK-1; EC 2.7.11.1; UniProt P51617) was first characterized in 1996 by Cao et al. in human monocytes as a proximal mediator of IL-1-driven signaling (1). Structurally, IRAK1 comprises an N-terminal death domain (DD), a central serine/threonine kinase domain, and a C-terminal proline/serine/threonine-rich (ProST) region (**Figure 1C**). This tripartite structure enables IRAK1 to facilitate different roles as a scaffold, signal amplifier, and phosphorylation-dependent activator within the Myddosome complex, a helical oligomeric signaling assembly that coordinates the receptor-proximal activation of IRAK4, IRAK1, and IRAK2 (12, 13).

IRAK1 activation in canonical innate immune signaling involves hierarchical phosphorylation initiated by IRAK4, leading to autophosphorylation that enables dissociation from the Myddosome, an oligomeric signaling complex that serves as a key platform for the transmission of immune signaling, interaction with TNF receptor-associated factor 6 (TRAF6), and activation of the NF-κB and mitogen-activated protein kinase (MAPK) pathways (2, 14). It is subsequently degraded via K48-linked ubiquitination, creating a self-limiting inflammatory response (15). Beyond canonical innate immune signaling, IRAK1 participates in non-canonical functions, including nuclear translocation, signal transducer and activator of transcription 3 (STAT3) phosphorylation, IRF7-dependent antiviral responses, and oncogenic transcriptional co-activation (16, 17). The regulatory mechanisms governing IRAK1 are predominantly encoded within its post-translational modification (PTM) landscape, particularly the spatial, temporal, and site-specific phosphorylation events that govern its activation state, protein interactions, and downstream signaling output (18, 19). Advances in mass spectrometry-based phosphoproteomics have enabled the detection of IRAK1 phosphorylation events at the system level, substantially expanding the known phosphosite repertoire beyond the few biochemically validated residues traditionally studied (20–22). However, these phosphoproteomic data remain fragmented across different studies, precluding the systematic identification of disease-specific phosphorylation signatures and druggable regulatory nodes. The functional significance of most observed phosphosites remains uncharacterized. While disease-specific phosphorylation signatures have been proposed as biomarkers, the lack of site-level functional annotation prevents mechanistic interpretation of IRAK1 dysregulation in autoimmune diseases and cancer.

This review focuses on systematically identifying the IRAK1 phosphosites that are most frequently observed and differentially regulated across diverse biological contexts. We discuss how the localization of these phosphosites within IRAK1 functional domains modulates regulatory mechanisms. Additionally, this review prioritizes high-confidence phosphosites as key targets for functional validation and therapeutic intervention. Existing phosphosite repositories aggregate detection events without contextual filtering or functional annotation. To overcome these limitations, we systematically integrated publicly available 246 IRAK1 phosphoproteomic datasets and applied detection frequency, domain distribution, and differential regulation as orthogonal prioritization criteria, establishing a data-driven framework to distinguish biologically meaningful IRAK1 phosphosites from technical artifacts and guide experimental validation. We further analyzed 213 profiling-based and 33 quantitative proteomic datasets to explore domain-resolved phosphorylation landscapes, cross-contextual detection frequencies, and co-regulated PTM interactions. We hypothesize that phosphosites detected across diverse biological contexts and showing differential regulation are more likely to encode regulatory functions than sites detected only in single studies or under basal conditions, and we use this as a criterion for prioritization. We also discuss the biological implications of dysregulated IRAK1 phosphorylation in autoimmune and malignant diseases and evaluate the current pharmacological strategies targeting IRAK1 kinase activity. Together, this integrated perspective provides a data-driven framework for translating phosphoproteomic discoveries into mechanistic insights and therapeutic opportunities.

## 2 Molecular architecture and cellular biology of IRAK1

### 2.1 Gene organization, expression, and subcellular localization

The human *IRAK1* gene is located on chromosome Xq28, spans approximately 9.4 kb of genomic DNA, and encodes a 712-amino-acid protein with a predicted molecular mass of approximately 76 kDa (23). IRAK1 is ubiquitously expressed, with the highest expression levels documented in immune-relevant tissues, including the pancreas, gastrointestinal tract, and brain, consistent with its central role in innate immune surveillance and host defense (24). Under basal conditions, IRAK1 localizes predominantly to the cytoplasm; however, activation-dependent nuclear translocation has been reported in multiple cell types, including epithelial and hematopoietic cells (25). Nuclear IRAK1 has been shown to interact with NF-κB-responsive promoter elements and transcriptional machinery, directly regulating inflammatory gene expression (17). Post-translational modifications, particularly phosphorylation and SUMOylation, have been implicated in the regulation of nucleocytoplasmic shuttling (26, 27). The ability of IRAK1 to occupy both the cytoplasmic and nuclear compartments in an activation state-dependent manner substantially expands the functional scope of IRAK1 beyond receptor-proximal signal transduction (25).

### 2.2 Domain architecture and functional determinants

IRAK1 exhibits a canonical tripartite domain structure that supports multifunctional regulation. IRAK1 generally comprises an N-terminal death domain (DD; ~1–103 aa), a ProST region (~104-198aa), a central kinase domain (~199-522aa) containing an activation loop (~364-388aa), and two C-terminal domains, C1 (~523-618aa) and C2 (~619-712aa) (6, 28). Alternative annotations have defined the death domain as residues 1-109, the ProST region as residues 110-211, the kinase domain as residues 212-536, and the C-terminal region as residues 537-712 (11). These discrepancies originate from the use of operational boundaries in deletion, biochemical, and structural studies rather than from fundamentally distinct protein architectures (6, 28). The death domain mediates homotypic protein-protein interactions, including the recruitment of IRAK1 to Myeloid differentiation primary response 88 (MyD88) at the activated receptor complex. Thr66 within the death domain is important for IRAK1 homodimerization and heterodimerization with other IRAK family members (29). Within the kinase domain, residues Lys239 and Asp340, located in the ATP-binding region, are essential for catalytic activity, and point mutations of either residue (K239S or D340N) completely reduce IRAK1 kinase activity (30, 31). The activation loop (~364-388aa) contains Thr209 and Thr387, both of which are phosphorylated by IRAK4 upon receptor engagement, resulting in the activation of IRAK1 kinase activity and subsequent IRAK1 auto-phosphorylation (3, 32, 33).

IRAK1 binds to the E3 ligase-associated adaptor TRAF6 through multiple regions of the protein, including the death domain, ProST region, and C-terminal C1 domain (34). Two regions have been implicated in the susceptibility of IRAK1 to degradation: a 186-amino-acid region located between the death domain and the kinase domain (i.e., spanning the ProST region) and a 30-amino-acid region at the C-terminal end of the kinase domain; deletion of either region renders IRAK1 resistant to degradation (30, 34). The kinase domain contains conserved structural features typical of serine/threonine kinases, including a glycine-rich ATP-binding loop near the N-terminus and a conserved catalytic aspartic acid residue located centrally within the domain (35). The ProST domain also functions as a molecular timer; progressive autophosphorylation within this region facilitates IRAK1 ubiquitination and proteasomal degradation, thereby coupling kinase activation to self-termination of signaling. This intrinsic negative regulatory mechanism ensures transient rather than constitutive activation under physiological conditions (11). Structural prediction models and existing crystallographic data indicate that the kinase domain adopts a canonical bi-lobed fold, with the N-lobe primarily involved in ATP binding and the C-lobe involved in substrate recognition and phosphotransfer (6).

## 3 Canonical activation mechanism: myddosome assembly and hierarchical phosphorylation

### 3.1 Myddosome complex formation and sequential IRAK recruitment

Upon binding of TLRs or IL-1Rs by pathogen-associated molecular patterns (PAMPs), damage-associated molecular patterns (DAMPs), or cytokine ligands (e.g., IL-1β, IL-18, IL-33), the cytosolic Toll/IL-1 receptor (TIR) domains of activated receptors recruit the adaptor protein MyD88 (36, 37). MyD88, through its death domain, initiates the sequential recruitment of IRAK4 and IRAK1/IRAK2, forming the supramolecular Myddosome complex in which the death domain assembles into a left-handed helical oligomeric structure with defined stoichiometry (12, 38). Within the Myddosome, IRAK4 occupies the innermost ring in close proximity to MyD88, thereby functioning as primary upstream activating kinase. IRAK4 initiates signaling by trans-phosphorylating IRAK1 at Thr209, inducing a conformational change that permits subsequent phosphorylation of Thr387 within the activation loop, leading to full catalytic activity. This phosphorylation event induces a conformational rearrangement in the kinase domain of IRAK1, releasing the activation loop from its autoinhibited conformation and enabling substrate engagement. Activated IRAK1 subsequently undergoes extensive autophosphorylation within the kinase region (Ser376, Ser384, Thr387, and Ser390), augmenting its catalytic activity and signaling for Myddosome dissociation (11).

### 3.2 IRAK1-TRAF6 interaction and downstream signal propagation

The interaction between IRAK1 and TRAF6 triggers an essential signaling cascade that connects the dissociation of the Myddosome to the activation of downstream kinases and transcription factors involved in the inflammatory response. Once the Myddosome dissociates, phosphorylated IRAK1 associates with the E3 ubiquitin ligase TRAF6 and the adaptor proteins TRAF6-binding domain-containing proteins, forming a secondary signaling complex at the plasma membrane. This IRAK1-TRAF6-TAB2 complex is subsequently released into the cytoplasm, where it activates transforming growth factor-beta-activated kinase 1 (TAK1), Inhibitor of KappaB Kinase (IKK), and stress-activated kinase cascades (39). This interaction stimulates TRAF6 E3 ligase activity, promoting the assembly of K63-linked polyubiquitin chains on TRAF6 and IRAK1 (40, 41). These non-degradative ubiquitin chains function as scaffolds for the TAK1-TAB2/TAB3 complex, enabling TAK1-mediated phosphorylation and activation of the IKK complex (IKKα/β/γ) and the MAPK cascade (Mitogen-activated protein kinase kinase (MKK)3/6-p38, MKK4/7- c-Jun N-terminal kinase (JNK), and Mitogen-activated protein kinase kinase (MEK)1/2- Extracellular signal-regulated kinase (ERK)) (42, 43). IKK activation leads to the phosphorylation and proteasomal degradation of IκB proteins, releasing NF-κB dimers for nuclear translocation and transcriptional activation of pro-inflammatory target genes encoding cytokines (IL-6, Tumor necrosis factor (TNF)-α, IL-12), chemokines, and adhesion molecules. Simultaneously, MAPK activation promotes activator protein 1 (AP-1)-dependent transcriptional responses and post-transcriptional regulation of cytokine mRNA stability. The net result is a coordinated and amplified innate immune gene expression program appropriate for the nature and magnitude of the initiating stimulus (44). Negative regulation of this signaling cascade is achieved through multiple convergent mechanisms: K48-linked ubiquitination of IRAK1 by the Skp1-Cullin1-F-box β-transducin repeat-containing protein (SCFβ-TrCP) E3 complex targets it for 26S proteasomal degradation; accumulation of IRAK-M (IRAK3) sequesters signaling-competent IRAK1/IRAK4 complexes; and induction of miR-146a post-transcriptionally suppresses IRAK1 and TRAF6 expression, collectively ensuring signal attenuation and prevention of chronic inflammatory activation (45).

## 4 IRAK1 in innate immune and non-canonical signaling pathways

### 4.1 TLR-mediated innate immune signaling

Within the TLR signaling network, IRAK1 operates as a non-redundant amplifier of MyD88-dependent pathway activation and propagates signals from all MyD88-dependent TLRs (TLR1, 2, 4, 5, 6, 7, 8, and 9) to downstream NF-κB and MAPK effectors (7). Genetic ablation of IRAK1 markedly impairs IL-1β-, IL-18-, and TLR-ligand-induced NF-κB activation and cytokine production (IL-6, TNF-α, IL-12), demonstrating its essential and non-compensable roles in these responses (46). In plasmacytoid dendritic cells (pDCs), IRAK1 plays a critical role in TLR7 and TLR9 signaling, where it is required for IRF7 phosphorylation and nuclear translocation, driving type I interferon (IFN-α/β) production, which is essential for antiviral immunity (16). This function differs from that of IRAK4, which primarily controls the NF-κB arm of TLR signaling, highlighting the pathway-level specialization within the IRAK kinase family.

### 4.2 IL-1 receptor family signaling

The IL-1 receptor (IL-1R) family, which comprises IL-1R1, IL-18R, IL-33R (ST2), and IL-36R, plays a pivotal role in innate immune responses by initiating signaling cascades that result in the expression of proinflammatory genes. These receptors consistently utilize the adaptor protein MyD88 to recruit and activate IRAKs, particularly IRAK4 and IRAK1, which are essential for downstream signaling events (47, 48). Upon ligand binding, MyD88 facilitates the assembly of the Myddosome complex, enabling IRAK4 to phosphorylate and activate IRAK1. Once activated, IRAK1 interacts with TRAF6, leading to the further activation of transcription factors, such as NF-κB and MAPKs, including JNK and p38, which promote the transcription of pro-inflammatory cytokines (49, 50). This implies a divergence in signaling downstream of IL-1R, where IRAK1 mediates both NF-κB-dependent and independent pathways. IL-18R signaling shares similar mechanisms, with MyD88-dependent recruitment of IRAK4 and IRAK1 leading to NF-κB and MAPK activation, which promotes IFN-γ production and increases Th1 responses (42). Post-translational modifications of IRAK1, such as phosphorylation and ubiquitination, modulate its stability and signaling capacity, affecting the duration and significance of the inflammatory response. IL-1R family signaling through MyD88 and IRAK4-IRAK1 is essential for initiating and regulating innate immune responses, with distinct receptor members directing specific immune outcomes through common downstream pathways.

### 4.3 Non-canonical and myddosome-independent functions

Recent research has shown that IRAK1 performs functions beyond its canonical role within the Myddosome and can be activated independently of traditional TLR/IL-1R signaling (51). IRAK1 can translocate to the nucleus and directly phosphorylate STAT3 at Ser727, thereby increasing STAT3 transcriptional activity in response to inflammatory stimuli (17, 52). Nevertheless, the degree to which the IRAK1-STAT3 axis functions independently of canonical Myddosome signaling and its contribution to distinct subcellular STAT3 pools, including mitochondrial STAT3, remain unclear.

IRAK1 also modulates the activity and stability of Monocyte Chemoattractant Protein-Induced Protein 1 (MCPIP1), also known as Regnase-1, which is encoded by the ZC3H12A gene and functions as an endoribonuclease that degrades transcripts encoding pro-inflammatory cytokines and other immune mediators (53). Upon TLR or IL-1R stimulation, IRAK1-dependent phosphorylation of Regnase-1 leads to its functional inactivation and subsequent regulation by the IKK complex and ubiquitin-proteasome system, facilitating the stabilization and accumulation of inflammatory mRNAs. Recent results have shown that IRAK1-dependent phosphorylation of Regnase-1 at Ser494 and Ser513 increases its interaction with 14-3–3 proteins and suppresses Regnase-1-mediated mRNA decay (54). Although IRAK1 kinase activity is essential for this process, it remains unclear whether IRAK1 directly phosphorylates these residues or acts through downstream kinases.

In antiviral signaling, IRAK1 effectively cooperates with viperin (RSAD2), a radical S-adenosylmethionine enzyme that catalyzes the formation of the antiviral nucleotide 3′-deoxy-3′, 4′-didehydrocytidine triphosphate (ddhCTP) (55). IRAK1 and TRAF6 enhance viperin activity, whereas viperin facilitates IRAK1 ubiquitination, thereby linking innate immune signaling to antiviral nucleotide metabolism (56). Thus, the IRAK1-viperin axis represents a non-canonical connection between innate immune signaling and metabolic antiviral defense. In addition to its established roles in innate immune signaling, dysregulated IRAK1 activity has been implicated in oncogenic signaling networks, including Janus kinase/signal transducer and activator of transcription (JAK/STAT3)-dependent pathways (57). Persistent IRAK1 activation contributes to inflammatory and pro-survival transcriptional programs through interactions with cytokine signaling pathways and STAT3 activation. However, the extent to which these effects reflect direct IRAK1-mediated phosphorylation of STAT3 versus indirect activation of the JAK/STAT pathway is context-dependent and should be mechanistically distinguished.

In addition to its established role in IL-1R and TLR signaling, IRAK1 has been implicated in the regulation of cytoskeletal signaling processes that influence cell adhesion, actin remodeling, and cell motility. Following IL-1 stimulation, IRAK-containing signaling complexes are recruited to nascent focal adhesions at the ruffled leading edge of spreading human fibroblasts, where they colocalize with MyD88, IL-1 receptor components, actin-capping proteins, and phosphorylated ERK. Depletion of actin-capping proteins interferes with the recruitment of these signaling components and attenuates IL-1-induced ERK activation and matrix metalloproteinase expression (58). These data support a model in which nascent focal adhesions function as spatially organized signaling platforms for IRAK1 activation. However, they did not demonstrate that IRAK1 directly regulates actin polymerization or independently drives focal adhesion assembly. Rather, the available evidence indicates that IRAK1 is positioned within the cytoskeletal signaling domains to facilitate localized inflammatory and ERK signaling.

IRAK1 can also link TLR signaling to the Rho-family GTPase Rac1. In macrophages stimulated with lipopolysaccharide, IRAK1 associates with and activates Rac1 through an N-terminal L167WPPPP motif, and this interaction contributes to NOX1 activation and reactive oxygen species (ROS) production (59). As Rac1 is a central regulator of lamellipodium formation, cell spreading, focal adhesion dynamics, and actin remodeling, this interaction provides a mechanistic link between IRAK1 signaling and cytoskeletal organization. Nevertheless, the primary study assessed Rac1-dependent oxidase activation rather than directly measuring actin architecture or focal adhesion turnover. Thus, the cytoskeletal consequences of the IRAK1-Rac1 interaction remain mechanistically plausible but are incompletely defined.

A related mechanism involves the RhoA/Rho-associated coiled-coil-containing protein kinase (ROCK)-ezrin pathway. In pulmonary alveolar epithelial cells, lipopolysaccharide (LPS) induces RhoA/ROCK-dependent phosphorylation and membrane translocation of ezrin, followed by the induction of ezrin association with MyD88 and IRAK1. Inhibition of ROCK or ezrin reduces the downstream activation of p38 and NF-κB and attenuates the production of inflammatory cytokines (60). These data place IRAK1 within an inflammatory signaling module that interfaces with the plasma membrane and actin cytoskeleton, although they do not establish IRAK1 as a direct mediator of ezrin-dependent cytoskeletal remodeling. IRAK1-dependent effects on cell migration and invasion have also been described in cancer models. However, these phenotypes frequently involve NF-κB- or STAT3-dependent transcription, epithelial-mesenchymal transition, and altered expression of adhesion molecules, rather than a demonstrated direct effect of IRAK1 on the cytoskeletal architecture (61). Therefore, impaired migration following IRAK1 depletion or inhibition should not be interpreted as evidence of a direct structural role for IRAK1 in the cytoskeleton.

More direct evidence for cytoskeletal scaffolding function comes from the IRAK1-PKCϵ-VASP pathway in macrophages. IRAK1 acts downstream of PKCϵ and upstream of vasodilator-stimulated phosphoprotein (VASP), forms a complex with both proteins, and contributes to PMA-induced VASP phosphorylation. Loss of IRAK1 attenuates macrophage migration, whereas the L167A/W168A mutation disrupts IRAK1 association with VASP, identifying the L167WPPPP motif as a functional Ena/VASP homology 1 (EVH1)-binding element (62). Subsequent biophysical and structural analyses showed that the VASP EVH1 domain preferentially binds the trans-isomer of the IRAK1 Trp168-Pro169 peptide bond and that cyclophilin A accelerates this interaction by catalyzing prolyl isomerization (63). Together, these studies support a cytoskeleton-associated scaffolding function for IRAK1 that is mechanistically distinct from its canonical role in propagating inflammatory receptor signaling.

## 5 Post-translational regulation of IRAK1: a coordinated modification network

### 5.1 Phosphorylation dynamics and functional hierarchy

As detailed in Section 3.1, IRAK1 activity is directed by a hierarchical phosphorylation cascade initiated by IRAK4-mediated trans-phosphorylation at Thr209, followed by autophosphorylation in the kinase domain. In addition to enhancing catalytic activity, these events promote dissociation from MyD88/Tollip and Myddosome release. TRAF6 engagement, mediated by the DD, ProST region, and C1 domain, remains unaffected by this phosphorylation status (11, 34). The functional consequences of phosphorylation are, however, site-specific. Phosphorylation at Thr209 and Thr387 is primarily activating, promoting catalytic competence and Myddosome release (11). Phosphorylation at Thr66 within the death domain influences IRAK1 dimerization and higher-order complex formation, as demonstrated by FRET-based analyses (29). The broader repertoire of phosphosites identified through global phosphoproteomics, many of which are mapped outside the kinase domain, likely encodes additional layers of stimulus-specific regulation awaiting functional characterization.

### 5.2 Dephosphorylation and regulation by phosphatases

Although kinase-mediated phosphorylation of IRAK1 has been extensively characterized, dephosphorylation remains underexplored, despite its major regulatory role. Protein phosphatase 2A (PP2A) is the most comprehensively studied phosphatase that targets IRAK1. The catalytic subunit of PP2A directly associates with IRAK1, and its enzymatic activity increases within 30 min of IL-1β stimulation (64). Pharmacological inhibition of PP2A with okadaic acid or siRNA-mediated depletion of its catalytic subunit resulted in elevated IRAK1 phosphorylation, enhanced Lys48-linked ubiquitination, and increased proteasomal degradation. These observations establish PP2A as a negative regulator of IRAK1 turnover. In this context, PP2A activation is driven by the IL-1β-induced neutral sphingomyelinase-2 (NSMase-2)/ceramide signaling axis. PP2A-mediated dephosphorylation stabilizes IRAK1, promotes its retention at the cell membrane, and is essential for maximal JNK activation. This positive feedback mechanism modulates the intensity of IL-1β response (64).

The IRAK1-specific regulatory function of PP2A is especially important, given that PP2A is broadly recognized as a negative regulator of inflammatory signaling, directly dephosphorylating RelA (65), IKKβ (66), and members of the MAPK family (67, 68). Therefore, the overall impact of PP2A on the TLR/IL-1 pathway output is dependent on the specific substrate. At the level of IRAK1, PP2A sustains signal transduction by preventing phosphorylation-dependent degradation, whereas at downstream effectors, it restricts NF-κB and MAPK activation. PP2A is not the sole phosphatase acting on IRAK1; both PP1 and PP2A can fully dephosphorylate IRAK1 *in vitro*, indicating that multiple phosphorylation sites are targeted (69). Furthermore, PP1γ restores hyperphosphorylated IRAK1 to its unmodified state in cells, an effect that is blocked by the PP1/PP2A inhibitor microcystin (70). Two important limitations remain: the specific IRAK1 residues dephosphorylated by PP2A have not been identified (69), and IL-1-stimulated IRAK1 activation is not reversed by dephosphorylation or deubiquitylation. These observations suggest that IRAK1 catalytic activation is induced allosterically through its interaction with IRAK4 rather than by covalent modification (70).

Dephosphorylation also modulates the activity of the E3 ligase that is responsible for IRAK1 modification. Pellino isoforms gain the capacity to catalyze Lys63-linked polyubiquitination of IRAK1 only after phosphorylation by IRAK1 or IRAK4 *in vitro*, and this activation is reversed upon dephosphorylation (18). Because Pellino can be phosphorylated at multiple sites, dephosphorylation of several residues is required for its complete inactivation. This requirement may prolong the ligase activity of Pellino E3, consequently sustaining IRAK1 ubiquitination *in vivo* (71). Another distinct regulatory mechanism involves the tyrosine phosphatase SHP-1, which binds directly to the IRAK1 kinase domain and inhibits its activation (71). This interaction is mediated by an evolutionarily conserved KTIM (kinase tyrosyl-based inhibitory) motif within the IRAK1 kinase domain (72). By inactivating IRAK1, SHP-1 suppresses TLR-mediated proinflammatory cytokine production through the inhibition of NF-κB and MAPK activation, while simultaneously promoting TLR- and RIG-I-dependent type I interferon production through MyD88-, TRIF-, and RIG-I-dependent pathways. Thus, IRAK1 inactivation can redirect rather than simply terminate downstream signaling. It remains unresolved whether SHP-1-mediated IRAK1 inhibition depends on its catalytic activity or is primarily mediated by its binding. IRAK1 is tyrosine-phosphorylated, and its intrinsic kinase activity is elevated in SHP-1-deficient macrophages, whereas the interferon-promoting effect of SHP-1 is attributed to direct kinase domain binding. Pathogens can exploit this regulatory mechanism; for instance, Leishmania infection increases SHP-1 binding to IRAK1, resulting in IRAK1 inactivation (72). Future studies investigating the specific phosphatases responsible for site-specific dephosphorylation of IRAK1 will be critical to fully understand the dynamic regulation of the activation cycle and signal termination within inflammatory signaling networks.

### 5.3 Ubiquitination: K63- and K48-linked pathways

IRAK1 is subject to two functionally distinct polyubiquitin chain modifications that determine diametrically opposed signaling outcomes. K63-linked ubiquitination, catalyzed primarily by TRAF6 in cooperation with the Ubiquitin-conjugating enzyme 13 - ubiquitin-conjugating enzyme E2 variant 1A (UBC13-UEV1A E2) conjugating complex, is a non-degradative modification that assembles signaling scaffolds. These K63-ubiquitin chains serve as docking platforms for the TAK1-TAB2/3 complex, enabling efficient signal relay to IKK and MAPK effectors (41). In contrast, K48-linked polyubiquitination, mediated by the SCFβ-TrCP E3 ligase complex following IRAK1 hyperphosphorylation, targets IRAK1 for proteasomal degradation (73). The phosphorylation state of the IRAK1 ProST region, particularly its PEST-like sequences (residues 117–133 and 153-180), has been proposed to promote efficient K48-ubiquitin chain assembly, mechanistically coupling signal activation to the initiation of negative feedback (15, 74). However, the precise phosphosite-lysine pairings remain undefined, and conflicting reports suggest that IRAK1 degradation may also proceed via K63-ubiquitination independent of the proteasome (7), leaving the proteolytic mechanism unresolved.

### 5.4 SUMOylation and PTM crosstalk

SUMOylation of IRAK1 has been documented as a PTM that contributes to nuclear accumulation and transcriptional regulatory functions (25). SUMO modification at lysine residues may compete with ubiquitination at the same sites, thereby modulating protein stability and extending IRAK1 nuclear residence time. This PTM competition adds another layer of regulatory control, potentially enabling context-dependent switching between cytoplasmic signaling and nuclear transcriptional functions. The integration of phosphorylation, K63/K48-ubiquitination, and SUMOylation constitutes a dynamic PTM code that encodes both the amplitude and subcellular localization of IRAK1 activity, and its full decipherment is a priority for future structural and biochemical investigations.

## 6 Phosphoproteomic landscape of IRAK1: systematic dataset integration

### 6.1 Overview of phosphoproteomic approaches to IRAK1

Mass spectrometry-based phosphoproteomics has greatly enhanced the ability to analyze phosphorylation networks on a system scale. However, the distributed nature of published datasets differing in cellular context, stimulation conditions, enrichment methodology, and temporal resolution has hindered the integration needed to fully understand the IRAK1 phosphorylation landscape. In most phosphoproteomic studies, IRAK1 sites are discovered incidentally as part of broader signaling research rather than being the primary focus, resulting in limited coverage of IRAK1 phosphopeptides and complicating the interpretation of specific sites (75, 76). To address this challenge, we systematically integrated publicly available human cellular phosphoproteomic datasets reporting IRAK1 phosphorylation using established phosphoproteomic data standards and quality metrics (77). Our integration included datasets from diverse biological conditions, including IL-1β and TLR ligand stimulation, cellular stress responses, malignant cell lines, primary immune cell populations, and patient-derived samples, providing cross-contextual insights into the IRAK1 phosphorylation landscape.

### 6.2 Integrated phosphosite mapping of IRAK1

A systematic PubMed search was performed using the query “phosphoproteomics OR phosphoproteome” restricted to human studies, excluding plant organisms and review articles. The dataset selection criteria included only mass spectrometry-based datasets published until May 2025 that reported IRAK1 phosphosites with class I confidence (localization probability >0.75 or A-score >13), consistent with community standards for high-confidence site assignment (77). This threshold minimizes false-positive site assignments arising from ambiguous peptide fragmentation. Studies that lacked site-level localization information or reported only ambiguous phosphopeptides were excluded. IRAK1 phosphosites were extracted and mapped to the canonical human IRAK1 sequence (UniProt P51617) using a standardized, in-house computational pipeline.

Experimental metadata, including cell type, disease condition, treatment, time point, and quantitation method (stable isotope labeling by amino acids in cell culture (SILAC), tandem mass tag (TMT), and label-free quantification), were aligned according to standard phosphoproteomics guidelines (77). The datasets were categorized into two types: (i) profiling datasets, which report qualitative IRAK1 phosphosite lists, and (ii) differential datasets, which report quantitative phosphorylation changes. For differential datasets, we applied the fold-change thresholds reported in the original study when available; in cases where thresholds were not provided, we applied ≥1.3-fold upregulation and ≤0.76-fold downregulation, consistent with standard phosphoproteomics guidelines (77), and required a false discovery rate (FDR) < 0.05 or equivalent statistical significance. Redundant phosphopeptides mapping to identical IRAK1 residues were consolidated while maintaining the biological context (cell type, stimulus, and time point) (**Supplementary Tables 1, S2**). Peptides with uncertain site localization (localization probability <0.75) were excluded, and non-human or off-target sequences were removed. Phosphosite domain annotations were assigned using the InterPro and Pfam databases, and sequence coverage with site density was visualized using the Sequence Coverage Visualizer (SCV) tool (78). The lollipop plots illustrating the phosphorylation pattern of IRAK1 phosphosites were generated using the R/Bioconductor package trackViewer (v1.47.0) (79).

### 6.3 IRAK1 phosphosite landscape across studies

**Figure 2:**
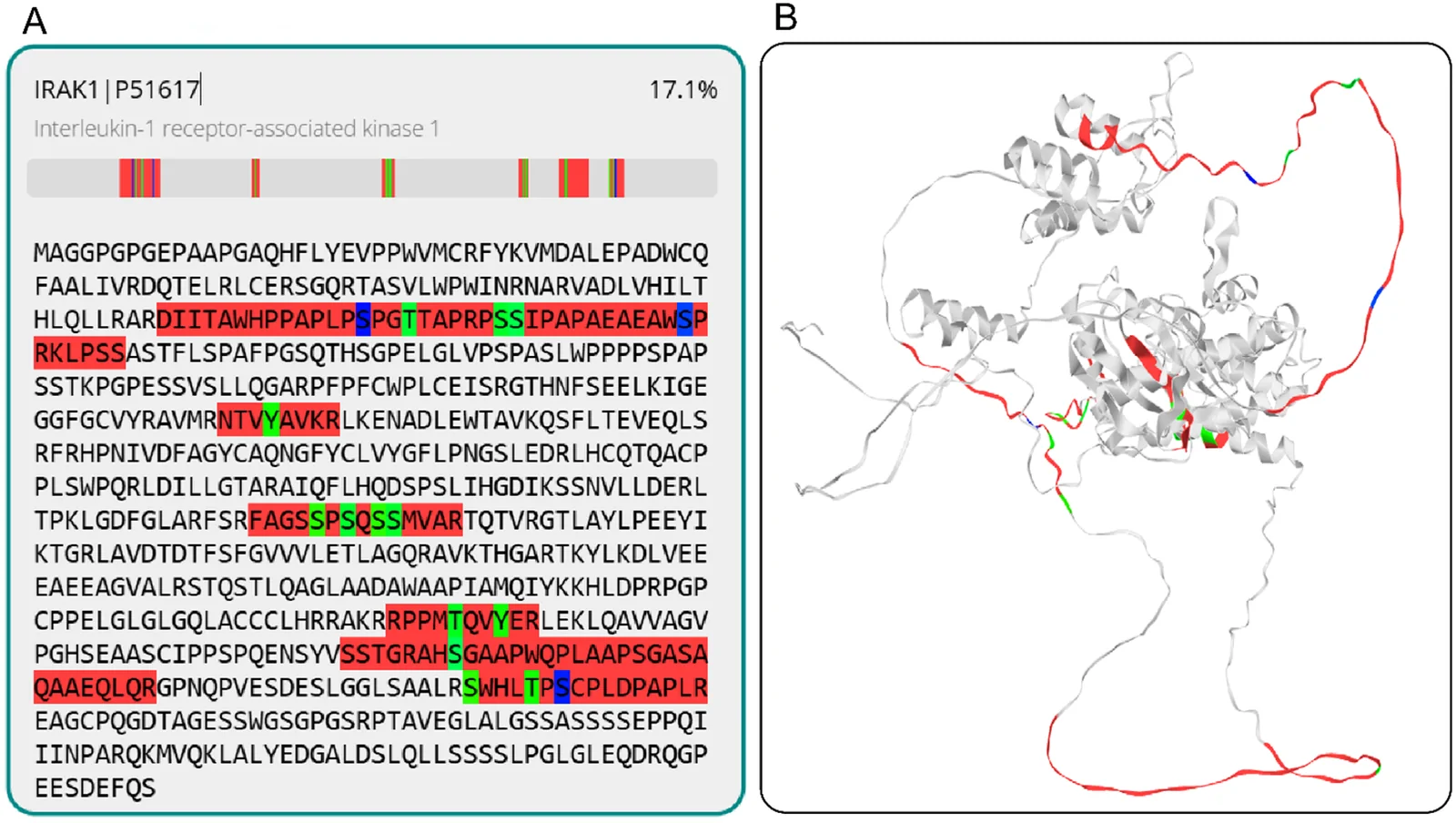
Sequence coverage and phosphosite mapping of IRAK1. **(A)** Linear sequence of IRAK1 showing peptide coverage identified in global human phosphoproteomic datasets. **(B)** AlphaFold-predicted three-dimensional structure of IRAK1 [UniProt ID: P51617; PDB ID: 6BFN] highlighting peptides detected in human cellular phosphoproteomic datasets, generated using the Sequence Coverage Visualizer (SCV; https://scv.lab.gy/). The sequence coverage is shown in red, Ser/Thr/Tyr phosphosites are indicated in green, and the predominant phosphosites are highlighted in blue.

The comprehensive integration of phosphoproteomic datasets resulted in 17.1% peptide coverage of the full-length IRAK1 protein sequence. Eighteen phosphosites were identified in the profiling datasets and 16 in the differential datasets, encompassing ten unique phosphopeptides within the same 17.1% of the IRAK1 sequence. The coverage of the death domain was limited (**Figure 2**), likely due to technical challenges in peptide recovery or low-abundance phosphorylation, which constrains the completeness of the phosphosite map. The detected peptides were primarily distributed across the serine/threonine kinase domain (residues 212-522) and C-terminal regulatory region (residues 523-712). In contrast, peptides mapping to the death domain (residues 1-106) were underrepresented, likely due to limited tryptic peptide recovery, low-abundance phosphorylation, or insufficient sampling of conditions where death domain phosphorylation is prominent. These distribution patterns are derived from the dataset and may be influenced by technical biases in peptide detection and the biological contexts represented in the literature.

**Figure 3:**
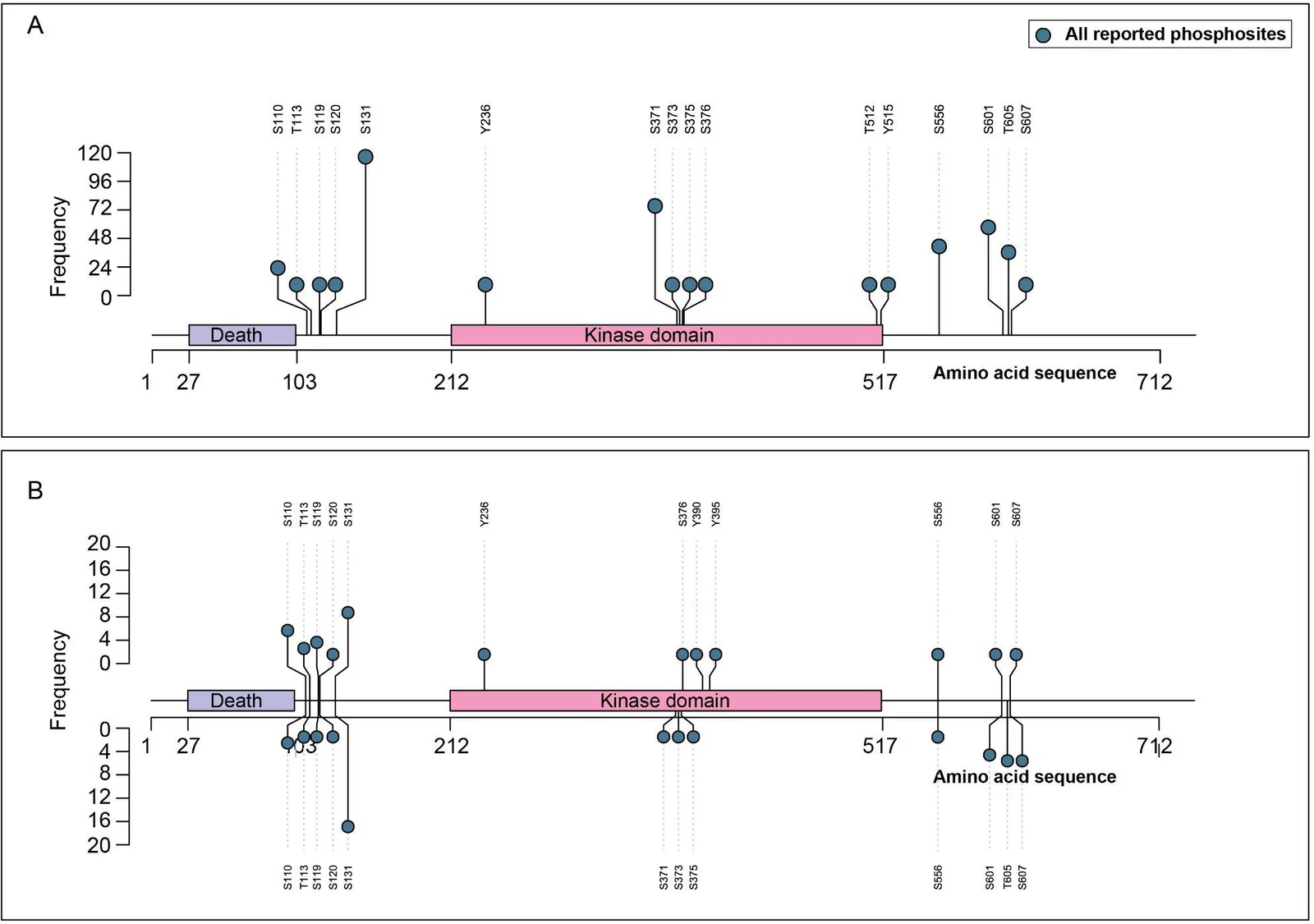
Lollipop plot representing differentially regulated IRAK1 phosphorylation sites. Lollipop plot visualization of IRAK1 class-I phosphosites across **(A)** profiling datasets and **(B)** differential datasets; schematic representation of IRAK1 class-I phosphosites mapped along the amino acid sequence, indicating the Death and Kinase domains. The y-axis represents the phosphosite frequency across the analyzed datasets. In panel **(B)**, phosphosites plotted above the x-axis represent positively co-regulated phosphosites, whereas those below the x-axis represent negatively co-regulated phosphosites. The y-axis indicates the frequency of phosphosite co-regulation across the datasets. The lollipop plots illustrating the phosphorylation pattern of IRAK1 phosphosites were generated using the R/Bioconductor package trackViewer (v1.47.0).

Across 213 profiling datasets and 33 differential datasets (derived from 50 and 6 PubMed-indexed studies, respectively), 18 and 16 unique IRAK1 phosphosites were identified. The detection frequency landscape was markedly nonuniform. In this integrated dataset, Ser131 was the most frequently detected site across both profiling (128 datasets) and differential (26 datasets) analyses, followed by Ser110 (26 profiling, 9 differential) and Ser607 (7 profiling, 7 differential). Sites with high profiling frequency but low differential frequency, such as S601, T605, S371, and S556, were detected consistently but showed limited evidence of dynamic regulation and were therefore assigned a lower priority for functional validation. These detection frequencies reflect the phosphosite abundance and technical accessibility under the observed conditions. These data are summarized in **Table 1** and illustrated as a lollipop plot of differential phosphosite frequency (**Figure 3**).

**Table 1:** Summary of IRAK1 phosphorylation sites: detection frequency and differential regulation across phosphoproteomic datasets.

Phosphosite	Domain	Detection frequency (profiling)	Detection frequency (differential)	Regulatory role	Validation method	References
Ser131	ProST Domain	128	26	Plays a critical regulatory role in the TLR signaling pathway, specifically for the induction of type I interferons	Immunoprecipitation, Mass spectrometry	(80–84)
Ser110	ProST Domain	26	9	Functional role not fully characterized; candidate for targeted validation	Mass spectrometry	(85)
Ser607	C-terminal Domain	7	7	Functional role not fully characterized; candidate for targeted validation	Mass spectrometry	(86)
Ser601	C-terminal Domain	61	6	Functional role not fully characterized; candidate for targeted validation	Mass spectrometry	(81, 85, 87, 88)
Thr605	C-terminal Domain	39	6	Functional role not fully characterized; candidate for targeted validation	Mass spectrometry	(88)
Ser119	ProST Domain	5	5	Functional role not fully characterized; candidate for targeted validation	Mass spectrometry	(86)
Thr113	ProST Domain	6	5	characterized; candidate for targeted validation	Mass spectrometry	(86, 89)
Ser120	ProST Domain	4	3	Functional role not fully characterized; candidate for targeted validation	Mass spectrometry	(86)
Ser371	Kinase Domain	81	2	Functional role not fully characterized; candidate for targeted validation	Mass spectrometry	(88, 90–92)
Ser373	Kinase Domain	1	1	Functional role not fully characterized; candidate for targeted validation	Mass spectrometry	(6)
Ser375	Kinase Domain	1	1	Functional role not fully characterized; candidate for targeted validation	Mass spectrometry	(86)
Ser376	Kinase Domain	2	2	Serves as a key indicator and component of IRAK1 kinase activation within the TLR signaling pathway	Mass spectrometry, Phospho-antibody, western blotting	(6, 93)
Ser556	C-terminal Domain	44	2	Functional role not fully characterized; candidate for targeted validation	Mass spectrometry	(81, 91, 94)

Phosphosites were mapped to the canonical human IRAK1 sequence (UniProt P51617). Detection frequency denotes the number of independent datasets in which each site was identified (profiling) or quantitatively regulated (differential analysis).

An exploratory hypothesis from these data is that the ProST-region phosphosites of IRAK1 may exhibit greater stimulus-dependent regulation than kinase domain sites, which require further statistical testing. This observation may reflect the highly transient, stimulus-coupled nature of kinase domain phosphorylation events that are difficult to capture at steady state by mass spectrometry, or alternatively, may indicate that ProST-region phosphorylation events are more durably regulated across diverse biological contexts than activation loop modifications. This distinction has important implications for prioritizing phosphosites in targeted validation studies.

### 6.4 Functional interpretation and knowledge gaps

Global phosphoproteomics has substantially increased the number of identified IRAK1 phosphosites; however, functional annotation of most sites remains limited. Thr209 and the ProST cluster (Ser376-Ser390) are the most thoroughly characterized, with roles in activation-loop priming, TRAF6 recruitment, and signal termination established through mutagenesis, phospho-specific antibody detection, and *in vitro* kinase assays (6, 11). In contrast, high-frequency sites, such as Ser131, which appears in 128 independent profiling datasets, have received minimal targeted functional investigation. Although preliminary evidence links Ser131 phosphorylation to IRAK1 activation (82), direct mechanistic validation, including the identification of upstream kinases, functional mutagenesis, and demonstration of regulatory consequences, is still lacking. Therefore, while frequent detection highlights Ser131 as a candidate for further study, its functional significance cannot be determined solely from the phosphoproteomic data. Ser131 resides within a Ser-Pro motif, and structural and biochemical studies suggest that this phosphorylated motif can bind to the prolyl isomerase Pin1 (71). However, the relevance of this interaction in living cells remains unexplored. Based on motif characteristics, proline-directed kinases, such as CDK, MAPK, or GSK3 family members, are plausible upstream regulators; however, this hypothesis has not been experimentally validated. The functional implications of Ser131 phosphorylation in specific disease contexts also require systematic characterization.

Ser607 was frequently detected as a phosphosite within the ProST region in both the profiling and differential datasets, indicating its potential relevance for further investigation. Phosphorylation sites in the ProST region beyond the canonical Ser376-Ser390 cluster may serve as novel regulatory nodes that modulate ubiquitination efficiency, proteasomal targeting, and the specificity of protein-protein interactions. The negative regulatory phosphosite at Thr100, identified through consensus motif analysis and validated by mutagenesis to disrupt the IRAK1-MyD88 interaction, further demonstrates that phosphorylation outside the kinase domain can exert significant regulatory effects that may be overlooked by analyses focused solely on the kinase domain (7, 33, 95).

Collectively, these findings underscore the significant gap between phosphosite identification and mechanistic understanding of phosphorylation. Addressing this gap requires integrative approaches that combine high-throughput phosphoproteomics with targeted functional validation. Such strategies should include phospho-specific mutagenesis, proximity ligation assays to map interactions, and phospho-flow cytometry for single-cell analysis to establish the causal relationships between IRAK1 phosphorylation events and downstream signaling outcomes.

## 7 Dysregulated IRAK1 phosphorylation in inflammatory disease and cancer

### 7.1 Chronic inflammation and autoimmunity

Persistent IRAK1 hyperactivation, arising from sustained phosphorylation and the failure of negative regulatory mechanisms, is a pathological driver of chronic inflammatory and autoimmune diseases. IRAK1 functions at the convergence point of TLR and IL-1R signaling networks, and its dysregulation amplifies NF-κB-driven pro-inflammatory cytokine production and type I interferon responses, both of which are hallmarks of chronic inflammatory pathology (96, 97).

Genetic studies have reported polymorphisms within the *IRAK1* locus, frequently found in linkage disequilibrium with the nearby *MECP2* gene on chromosome Xq28, as variants that increase susceptibility to systemic lupus erythematosus (SLE) and rheumatoid arthritis (RA) in various populations (98–100). Recently, IRAK1 variants have been associated with differential TLR7/9 signaling activity and altered IFN-α production in SLE cohorts, providing mechanistic plausibility for their disease-modifying effects. In RA, IRAK1 is prominently expressed and functionally active in synovial fibroblasts, where it drives IL-1β-dependent production of neutrophil chemoattractants and contributes to joint inflammation. Although elevated phospho-IRAK1 levels have been consistently reported in SLE and RA, current research focuses on the overall phosphorylation status rather than individual phosphosites. Site-resolved data linking specific residues to autoimmune pathology remain an area of investigation (101, 102).

### 7.2 Oncogenic IRAK1 signaling

In malignant contexts, IRAK1 transitions from a transiently activated immune mediator to a constitutively active, oncogenic driver. Overexpression, gain-of-function mutations, and loss of endogenous negative regulators (e.g., miR-146a, A20/TNFAIP3) contribute to aberrant IRAK1 phosphorylation, which sustains NF-κB and MAPK signaling in tumor cells, independent of extracellular ligand stimulation (103, 104). This constitutive signaling promotes cellular proliferation, survival, immune evasion, and therapeutic resistance. In hematologic malignancies, IRAK1 hyperactivation has been documented in myelodysplastic syndrome (MDS) and acute myeloid leukemia (AML), where it drives autocrine IL-1/IL-6 signaling that constitutively phosphorylates STAT3, supporting leukemic cell survival (105–107). Site-specific evidence supports this activation process. Phosphorylation at Thr209, the same IRAK4-dependent activation-loop site described in Section 3.1, has been used as a direct biomarker of IRAK1 activity in T-cell acute lymphoblastic leukemia (T-ALL), where it was detected in the majority of leukemic cell lines examined (108). Dual IRAK1/IRAK4 inhibition has been reported to induce apoptosis in AML blasts with reduced toxicity to normal hematopoietic progenitors relative to leukemic cells in preclinical models (109, 110), suggesting selective therapeutic targeting.

**Figure 4:**
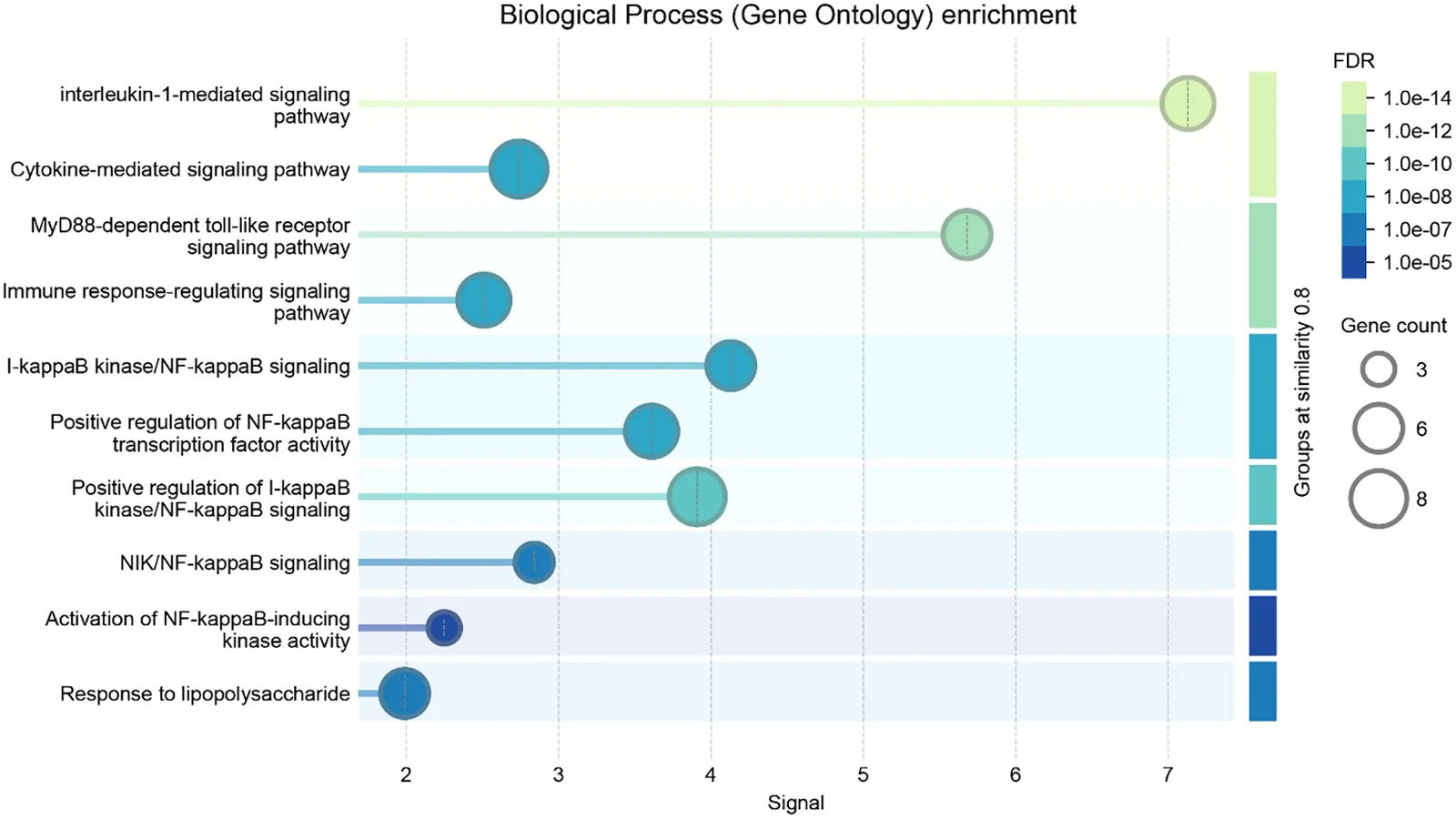
Gene ontology (GO) enrichment analysis of IRAK1-associated biological processes. GO enrichment was performed using the STRING database (v12.0) based on the IRAK1 protein-protein interaction network and the integrated GO enrichment pipeline. Statistical significance was assessed using the FDR, with GO terms having FDR < 0.05 considered significant. The x-axis represents the enrichment strength. The bubble size corresponds exclusively to the number of genes associated with each GO term. The bubble color indicates the FDR, with darker colors representing higher statistical significance. Functionally related GO terms were clustered based on semantic similarity (threshold: 0.8) to facilitate interpretation.

In solid tumors, such as triple-negative breast cancer (TNBC), IRAK1-driven NF-κB/STAT3 co-activation promotes metastatic gene programs and chemotherapy resistance. Inhibiting IRAK1 with pacritinib decreases STAT3 phosphorylation and enhances the sensitivity of TNBC cells to standard treatments (111–113). IRAK1 contributes to the pathogenesis of pancreatic ductal adenocarcinoma (PDAC). The tumor suppressor BRCA1-associated protein 1 (BAP1), frequently deleted in chronic pancreatitis-driven PDAC, normally binds to IRAK1 and restrains its interaction with IRAK4. Loss of BAP1 permits sustained IRAK4-mediated IRAK1 phosphorylation at Thr209 and downstream NF-κB activation, driving tumor cell proliferation, migration, and invasion. Consistent with this, IRAK1 is significantly upregulated in PDAC tumor tissues relative to adjacent peritumoral tissues and correlates with reduced progression-free survival. Moreover, dual pharmacological inhibition of IRAK1/IRAK4 suppressed NF-κB signaling in BAP1-deficient PDAC models. IRAK1 also shapes the tumor microenvironment through NF-κB-driven production of pro-tumorigenic cytokines and chemokines (e.g., IL-6, IL-8, and CCL2), which recruit immunosuppressive myeloid cells and promote angiogenesis (114, 115). In gastric cancer, IRAK1 overexpression correlates with poor prognosis and drives tumor cell proliferation, invasion, and EMT via the PI3K/AKT/mTOR signaling pathway. IRAK1 further promotes an immunosuppressive microenvironment by inducing tumor cell IL-8 secretion, which activates JAK2/STAT3 signaling in tumor-associated macrophages to drive M2-like polarization (116). IRAK1 effect on macrophage polarization is context-dependent. In colorectal polyps, IRAK1 loss via miR-146a-5p-mediated suppression attenuates pro-inflammatory M1 polarization, whereas in gastric cancer, IRAK1 drives immunosuppressive M2 polarization, highlighting the divergent roles of macrophage polarization in the tumor microenvironment (116, 117). The K63-ubiquitin scaffold function of IRAK1-associated complexes, involving RNF31 and TRAF6, reinforces sustained signaling by stabilizing the pro-tumorigenic transcriptional apparatus (118, 119). Collectively, these findings position IRAK1 at the interface of inflammation and oncogenesis, acting through core innate immune signaling pathways, such as MyD88-dependent TLR and NF-κB signaling (**Figure 4**), influencing both cell-intrinsic tumorigenic programs and the pro-tumorigenic microenvironment.

### 7.3 IRAK1 in sepsis and viral hyperinflammation.

Excessive IRAK1 activation also contributes to cytokine storms in sepsis and severe viral infections. TLR8-mediated recognition of GU-rich single-stranded RNA, including SARS-CoV-2- and HIV-1-derived sequences, drives rapid IRAK1 phosphorylation and ubiquitination, recruiting the TAK1 complex and triggering NOD-, LRR-, and pyrin domain-containing protein 3 (NLRP3) inflammasome assembly and downstream release of IL-1β, TNF, and IL-6 from macrophages. Pharmacological inhibition of IRAK1 with Pacritinib blocks the TLR8-IRAK1-TAK1 axis, attenuating the proinflammatory cytokine response independent of active viral infection, suggesting a potential therapeutic strategy for sepsis-associated hyperinflammation and long COVID (120–122). **Table 2** summarizes representative IRAK1-associated pathologies, mechanistic evidence, and experimental models in these disease contexts.

**Table 2:** IRAK1-associated pathologies, mechanistic evidence, and representative disease models.

Disease/ condition	Mechanistic insight	Key evidence	Experimental models	Key references
Systemic Lupus Erythematosus (SLE)	IRAK1 polymorphisms amplify TLR7/9-driven type I IFN production; promotes loss of immune tolerance	IRAK1 variants linked to elevated IRF7 phosphorylation and IFN-α; SNPs in IRAK1-MECP2 locus associated with SLE susceptibility	PBMC from SLE patients; murine lupus models	(123)
Rheumatoid Arthritis (RA)	IRAK1 genetic variants are associated with RA susceptibility	rs3027898 polymorphism distribution differs significantly between RA patients and controls	Case-control genetic cohort	(98)
Myelodysplastic Syndrome (MDS)	Constitutive IRAK1 activation sustains aberrant cytokine signaling and promotes clonal expansion	Overexpressed IRAK1 drives autocrine IL-6/IL-1; IRAK1 inhibition reduces colony formation	MDS patient-derived cells; *in vitro* kinase assays	(107)
Acute Myeloid Leukemia (AML)	IRAK1 hyperphosphorylation supports leukemic cell survival	IRAK1 overexpressed in AML; genetic knockdown and pacritinib reduce leukemia burden in xenografts	AML cell lines (MOLM-14, THP-1); patient-derived xenografts	(105)
Triple-Negative Breast Cancer (TNBC)	IRAK1-driven NF-κB and STAT3 signaling promotes metastasis and chemoresistance	IRAK1 knockdown sensitizes to paclitaxel; IRAK1-NF-κB-STAT3 axis drives metastasis	MDA-MB-231, SUM149 cell lines; xenograft models	(112)
Pancreatic Adenocarcinoma	IRAK1 sustains inflammatory tumor microenvironment and survival signaling	IRAK1 (and Thr209 phosphorylation) significantly upregulated in PDAC vs. peritumoral tissue; linked to reduced progression-free survival	PDAC tumor tissue; *in vivo*/*in vitro* PDAC models	(124)
Sepsis/SARS-CoV-2	Excessive TLR8-IRAK1 activation drives NLRP3 inflammasome assembly and cytokine storm	Pacritinib inhibits IRAK1 phosphorylation/ubiquitination, blocking TAK1 recruitment, NLRP3 assembly, and IL-1β/TNF/IL-6 release	Human primary macrophages	(122)
Gastric Cancer (GC)	IRAK1 overexpression drives GC progression via PI3K/AKT/mTOR signaling and induces immunosuppressive TAM M2 polarization via IL-8/JAK2/STAT3	IRAK1 expression correlates with GC progression and poor survival; IRAK1 knockout/overexpression confirms effects on proliferation, invasion, and EMT	Patient GC tissue; CRISPR/Cas9 IRAK1-KO and IRAK1-overexpressing GC cell lines; *in vivo* xenografts	(116)

## 8 Therapeutic targeting of IRAK1: pharmacological and endogenous strategies

### 8.1 Rationale for IRAK1-directed therapeutic intervention

The central positioning of IRAK1 at the convergence of innate immune, inflammatory, and oncogenic signaling pathways establishes a compelling therapeutic rationale for pharmacological inhibition. Unlike constitutively active kinases driven by somatic mutations, IRAK1 hyperactivation in most pathological contexts arises from dysregulated phosphorylation and loss of negative regulatory checkpoints, preserving the kinase domain as a structurally intact target for drug therapy (111, 125, 126). Multiple pharmacological approaches have been developed, including direct ATP-competitive kinase inhibition, covalent active-site targeting, redox-dependent enzymatic disruption, and augmentation of endogenous negative regulatory mechanisms, each with distinct selectivity and mechanistic profiles. The mechanisms of ATP competition, covalent interactions, redox dependency, and endogenous regulation are summarized. Information regarding the target sites was derived from the reported biochemical and structural data, as presented in **Table 3**.

**Table 3:** Pharmacological and endogenous regulators targeting IRAK1: mechanisms, target sites and disease indications.

Inhibitor/regulator	Class	Mechanism of action	IRAK1 target site/pathway	IRAK family selectivity	Disease indication	References
Pacritinib	ATP-competitive inhibitor	Binds IRAK1 catalytic domain; inhibits autophosphorylation and ubiquitination; blocks NF-κB and NLRP3 priming	Kinase domain; IRAK1-TRAF6-NF-κB axis	IRAK1-preferential, ~30-fold selectivity over IRAK4	Myelofibrosis, MDS, AML, TNBC, COVID-19	(112, 122, 127)
1, 4-Naphthoquinone	Redox-dependent small molecule	Inhibits IRAK1 catalytic activity via redox interference; impairs MyD88 complex assembly; reduces K63-ubiquitination of TRAF6	Catalytic cysteine; IRAK1-TAK1-IKK axis	IRAK1-directed via redox mechanism; full IRAK family selectivity profiling not yet characterized	Inflammatory diseases; IRAK1-dependent tumors	(130, 131)
JH-X-119-01	Covalent irreversible inhibitor	Forms stable covalent bond at cysteine adjacent to active site; sustains kinase inhibition post-washout; selective for MyD88-mutant contexts	IRAK1 active site cysteine residue	Highly IRAK1-selective; covalent binding at Cys302, unique to IRAK1 active site; IRAK4-sparing by mechanism	B-cell lymphoma (MyD88 L265P), sepsis	(128, 129)
Rosoxacin (Acrosoxacin)	Quinolone-derived reversible inhibitor	Blocks IRAK1 phosphorylation and TRAF6 recruitment; attenuates NF-κB-mediated survival and inflammatory signaling	IRAK1 kinase domain; TRAF6 interaction interface	IRAK1-selective based on screening	Autoimmune/inflammatory diseases; infectious disease	(132)
β-TrCP (SCF E3 ligase)	Endogenous negative regulator	Mediates K48-linked polyubiquitination of activated, hyperphosphorylated IRAK1; targets for 26S proteasomal degradation	ProST domain phospho-degron; K48-ubiquitination sites	Substrate-selective for hyperphosphorylated IRAK1. does not equivalently regulate other IRAK family members	Physiological signal termination; prevents chronic NF-κB	(73)
miR-146a	Post-transcriptional regulator	Binds 3’ UTR of IRAK1 mRNA; suppresses IRAK1 translation; negative feedback loop attenuates TLR/IL-1R-NF-κB signaling	IRAK1 mRNA (3’ UTR); translational suppression	mRNA-level selectivity for IRAK1 via 3’ UTR targeting; IRAK2, IRAK3, and IRAK4 mRNAs not equivalently targeted	SLE, RA, chronic inflammation; tumor suppression	(45, 136)
R835 (prodrug: R289)	Dual IRAK1/IRAK4 ATP-competitive inhibitor	Blocks TLR- and IL-1R-induced cytokine production, demonstrated proof-of-mechanism by suppressing LPS-induced serum cytokines in healthy volunteers in Phase 1	Kinase domain; dual IRAK1/IRAK4-TLR/IL-1R-NF-κB and NLRP3 axis	Dual IRAK1/IRAK4 inhibitor, selective over IRAK2 and IRAK3	Rheumatoid arthritis, SLE, gout, psoriasis, IBD (preclinical)	(144)
A34	ATP-competitive selective IRAK1 inhibitor	Binds IRAK1 ATP-binding pocket with high affinity (IC50 10.6 nM), inhibits IRAK1 autophosphorylation and downstream NF-κB signaling	Kinase domain ATP-binding pocket; IRAK1-NF-κB axis	Highly IRAK1-selective exceptional selectivity over 215 kinases including IRAK4	Hepatocellular carcinoma (HCC); preclinical stage	(143)
JNJ-1013 (Degrader-3)	IRAK1 degrader/Proteolysis-targeting chimera (PROTAC)	Induces IRAK1 protein degradation via the ubiquitin-proteasome system, thereby suppressing IRAK1 signaling and also eliminating scaffolding function	Targets IRAK1 protein; inhibits downstream IL-1R/TLR signaling and MyD88-dependent signaling	Selective for IRAK1 over off-targets; reported as highly potent and selective	MyD88-mutant ABC DLBCL/B-cell lymphoma	(141)
Compound 27	IRAK1/4/pan-FLT3 kinase inhibitor	Potent inhibition of IRAK1, IRAK4, and FLT3 kinase activity	IRAK1/IRAK4 and FLT3 signaling pathways	Selective for IRAK1, IRAK4, and FLT3; reduced hERG block reported	Acute myeloid leukemia (AML)	(146)
UR241-2	IRAK1/4 inhibitor	Blocks IL-1/IRAK1/4 signaling; reduces NF-κB activation and p65/p38 phosphorylation	IL-1 signaling axis upstream of IRAK1/4, affecting LSC survival	IRAK1/4-selective inhibitor	Acute myeloid leukemia (AML), including diagnosis and relapse LSCs	(147)
KME-2780	Dual IRAK1/IRAK4 ATP-competitive inhibitor	Suppresses TLR2-mediated NF-κB activation, IRAK1 autophosphorylation, JAK-STAT signaling, and LSPC function	Kinase domain; dual IRAK1/IRAK4-NF-κB, MAPK, and JAK-STAT axes	Dual IRAK1/IRAK4 inhibitor, selective over IRAK2 and IRAK3	MDS, AML (including FLT3-mutant); preclinical stage, warrants clinical investigation	(109)
KME-0584	Triple IRAK1/IRAK4/pan-FLT3 ATP-competitive inhibitor	ATP-competitive triple inhibitor of IRAK1, IRAK4, and pan-FLT3 that blocks NF-κB, JAK-STAT, and FLT3-driven survival signaling	Kinase domain; IRAK1/IRAK4-NF-κB, MAPK, and JAK-STAT axes; FLT3-driven survival signaling	Dual IRAK1/IRAK4 inhibitor with pan-FLT3 activity	Relapsed/refractory AML and high-risk MDS, including HMA+venetoclax-resistant patients; clinical study planned (Kurome Therapeutics)	(148)
Tollip (Toll-interacting protein)	Endogenous negative regulator/adaptor protein	Associates with the death and kinase domain of IRAK1 via its CUE domain, maintaining IRAK1 in a catalytically inhibited state and preventing autophosphorylation, upon TLR/IL-1R activation, IRAK1 phosphorylates Tollip, weakening the IRAK1-Tollip interaction and facilitating IRAK1 release to the Myddosome	IRAK1 death domain and kinase domain; TLR/IL-1R-MyD88-IRAK1 axis	Primarily regulates IRAK1 (and presumably IRAK2)	Physiological signal regulation; dysregulation implicated in chronic inflammation and endotoxin tolerance	(137)
Pellino proteins (Pellino-1, Pellino-2, Pellino-3)	Endogenous E3 ubiquitin ligases/IRAK1 ubiquitination regulators	Pellino-1 and Pellino-2 catalyze K63-linked polyubiquitination of IRAK1 via their C-terminal RING-like domain, promoting downstream NF-κB and MAPK activation; Pellino-3b acts as a negative regulator, with K63-ubiquitination of IRAK1 stabilizing its retention at the Myddosome and attenuating downstream signaling	Phosphothreonine residues on IRAK1 (FHA-mediated recognition); IRAK1 K63-ubiquitination sites; NF-κB and MAPK axes	Pellino-2 shows preferential interaction with phosphorylated IRAK1 via FHA domain	Physiological innate immune regulation; Pellino dysregulation implicated in inflammatory diseases and NLRP3 inflammasome-driven pathologies	(138, 139)
SOCS1 (Suppressor of Cytokine Signaling 1)	Endogenous negative regulator/E3 ubiquitin ligase adaptor	Induced as part of a negative feedback loop following TLR activation, promotes ubiquitin-mediated proteasomal degradation of IRAK1, thereby suppressing NF-κB activation and downstream inflammatory cytokine production.	IRAK1 (via SH2 domain interaction); TLR4-MyD88-IRAK1-NF-κB axis	Targets IRAK1 directly; broader TLR signaling inhibition via MAL/TIRAP and NF-κB p65	Physiological negative feedback regulation; dysregulation linked to chronic inflammatory diseases, autoimmunity, and cancer	(140)
HS-243	Dual IRAK1/IRAK4 ATP-competitive inhibitor	Binds the IRAK1 ATP-binding pocket (IC50–24 nM); inhibits IRAK1 kinase activity and downstream NF-κB and MAPK signaling; suppresses LPS-induced cytokine production in RA fibroblast-like synoviocytes	Kinase domain ATP-binding pocket; IRAK1/IRAK4-NF-κB and MAPK axis	Dual IRAK1/IRAK4 inhibitor	Rheumatoid arthritis (RA), preclinical; inflammatory diseases	(142)

### 8.2 Pacritinib: multikinase inhibition with IRAK1 selectivity

Pacritinib is an orally bioavailable, ATP-competitive multikinase inhibitor with high-affinity targets, including JAK2, FLT3, and IRAK1, and is FDA-approved for the treatment of myelofibrosis (127). Within the IRAK1 signaling axis, pacritinib binds to the IRAK1 kinase domain and inhibits autophosphorylation, thereby blocking TRAF6 engagement and downstream NF-κB and MAPK pathway activation. Pacritinib also suppresses NLRP3 inflammasome priming by reducing NF-κB-driven transcription of pro-IL-1β and NLRP3 components, attenuating the two-signal requirement for inflammasome activation (122). In the oncological context, pacritinib has demonstrated efficacy in IRAK1-overexpressing TNBC models by reducing IRAK1-NF-κB-STAT3 transcriptional programs and reversing chemoresistance (112). Emerging evidence supports its utility in hyperinflammatory infectious contexts, including HIV-1 and SARS-CoV-2 infections, where TLR8-IRAK1-NLRP3 axis hyperactivation contributes to immunopathology (122). The dual JAK2/IRAK1 inhibitory profile of pacritinib is particularly advantageous in hematologic diseases in which both pathways are concurrently dysregulated.

### 8.3 JH-X-119-01: covalent irreversible IRAK1 inhibition

JH-X-119–01 represents a mechanistically unique strategy for inhibiting IRAK1 using structure-guided covalent chemistry to achieve a prolonged and highly selective kinase blockade. Developed through the optimization of the dual IRAK1/IRAK4 inhibitor THZ-2-118, JH-X-119–01 targets a cysteine residue adjacent to the IRAK1 active site, forming an irreversible covalent adduct that maintains kinase inhibition after compound washout (128). This pharmacological irreversibility offers the potential for prolonged target engagement relative to reversible inhibitors, which may be advantageous in disease contexts characterized by rapid IRAK1 reactivation. In MyD88-mutant B-cell lymphoma models, JH-X-119–01 showed selective efficacy, as IRAK1 is a crucial downstream component of the constantly active MyD88(L265P) oncoprotein. Preclinical research has indicated anti-proliferative and pro-apoptotic effects in lymphoma cell lines and suppression of inflammatory cytokine production in models relevant to sepsis, indicating its potential use in oncological and inflammatory conditions (129).

### 8.4 Redox-mediated inhibition: 1, 4-Naphthoquinone

1, 4-Naphthoquinone inhibits IRAK1 kinase activity through a redox-dependent mechanism that interferes with ATP-binding site function and induces conformational disruption of the catalytic domain (130). This inhibition damages IRAK1 autophosphorylation, blocks MyD88 complex action, and reduces TRAF6 K63-ubiquitination and downstream TAK1-IKK-NF-κB activation. The redox-dependent mechanism offers chemical tractability for enhancing selectivity and potency; however, off-target redox effects on non-IRAK1 cysteine-containing proteins remain a potential risk that requires further pharmacological evaluation (131).

### 8.5 Selective kinase activity inhibition

Rosoxacin (Acrosoxacin/Eradacil), a quinolone-class antibacterial agent, was identified as a selective inhibitor of IRAK1 kinase through phenotypic and target-based screening approaches. Rosoxacin inhibits IRAK1 phosphorylation and disrupts the IRAK1-TRAF6 interaction, attenuating NF-κB-mediated inflammatory and pro-survival transcriptional output. Its established clinical safety profile as an antimicrobial agent provides a potential translational advantage for repositioning in IRAK1-dependent inflammatory indications, although selectivity profiling and *in vivo* efficacy in inflammatory disease models require further investigation (132).

Natural compounds and a few dietary bioactive molecules have also been identified as potent endogenous modulators of IRAK1-mediated signaling. For example, thymoquinone, a primary volatile constituent of *Nigella sativa*, demonstrates significant anti-inflammatory and protective effects in murine models of hepatitis and gastritis by directly suppressing IRAK1 kinase activity and blocking the downstream NF-κB and AP-1 pathways (133). Similarly, the xanthone derivative mangiferin alleviates colitis symptoms by attenuating IRAK1 phosphorylation and inhibiting NF-κB and MAPK (134). Additionally, the dietary flavonoid kaempferol targets upstream kinases, including Src, Syk, IRAK1, and IRAK4, effectively reducing the production of inflammatory mediators and downregulating macrophage activation (135). These observations highlight the versatility of targeting IRAK1 using both synthetic pharmacology and natural modulators to control hyper-inflammatory signaling networks.

### 8.6 Endogenous negative regulatory mechanisms

Five endogenous mechanisms represent natural templates for therapeutic amplification: (i) β-TrCP-mediated degradation (ii) miR-146a-dependent translational suppression, (iii) Tollip-mediated pre-activation inhibition, (iv) Pellino protein-dependent ubiquitination control, and (v) SOCS1-mediated feedback suppression.

**Figure 5:**
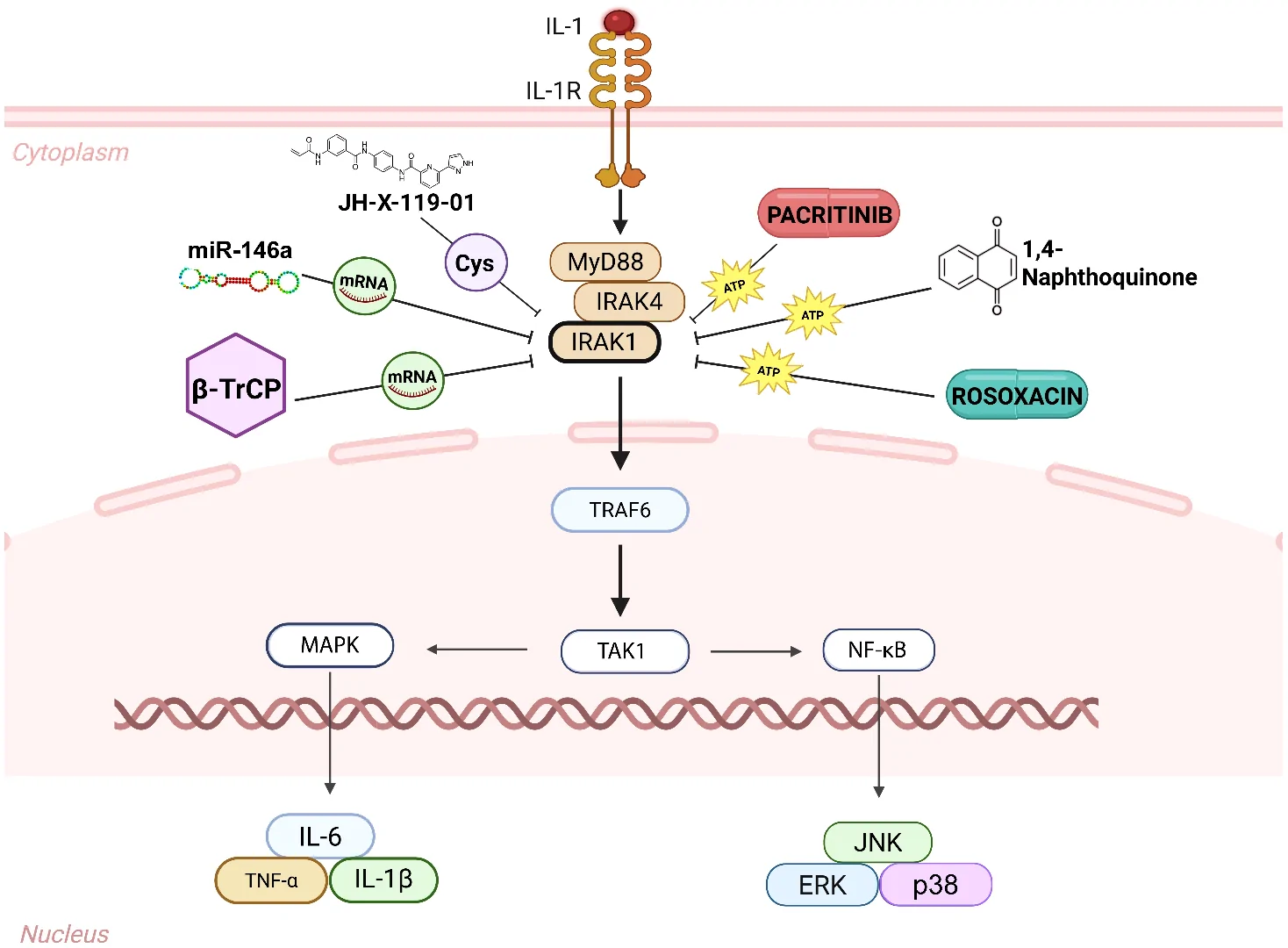
Regulation and inhibition of IRAK1 signaling in the IL-1R/TLR pathway. This image depicts the molecular mechanisms controlling IRAK1 signaling within the interleukin-1 receptor (IL-1R) and Toll-like receptor (TLR) pathways. The schematic illustrates key pharmacological inhibitors of IRAK1 signaling, including Pacritinib, Rosoxacin, JH-X-119-01, 1, 4-Napthoquinone, β-TrCP and miR-146a. These compounds inhibit IRAK1 kinase activity or IRAK1/IRAK4 signaling, thereby reducing downstream inflammatory responses. IL-1R, interleukin-1 receptor; TLR, Toll-like receptor; MyD88, myeloid differentiation primary response protein 88; IRAK1, interleukin-1 receptor-associated kinase 1; IRAK4, interleukin-1 receptor-associated kinase 4; TRAF6, tumor necrosis factor receptor-associated factor 6; TAK1, transforming growth factor-β-activated kinase 1; IKK, IκB kinase; NF-κB, nuclear factor-κB; MAPK, mitogen-activated protein kinase. Image generated using Biorender (2023) (https://www.biorender.com/). Created in BioRender; Source: https://BioRender.com/j4je8xg.

The SCFβ-TrCP E3 complex mediates K48-linked polyubiquitination of hyperphosphorylated IRAK1, targeting it for proteasomal degradation, thereby attenuating TLR/IL-1R signaling (73). Developing therapeutic targets to enhance β-TrCP recognition of phospho-IRAK1, for example, through phospho-mimetic degron-based small molecules, represents a novel approach to accelerate IRAK1 degradation in hyperinflammatory conditions. miR-146a functions as a post-transcriptional feedback regulator by binding to complementary sequences in the 3’ untranslated region (3’ UTR) of IRAK1 mRNA, suppressing IRAK1 translation, and reducing protein levels (45). Dual-luciferase reporter assays directly confirmed IRAK1 as a target of miR-146a-5p, with miR-146a-5p loss driving IRAK1-NF-κB-dependent M1 macrophage polarization and proinflammatory cytokine production in colorectal polyp tissue, an effect reversible by IRAK1 silencing (117). In autoimmune and inflammatory diseases characterized by reduced miR-146a expression (e.g., SLE, RA, and inflammatory bowel disease), restoration of miR-146a-mediated IRAK1 suppression through miRNA mimetics or delivery strategies represents a therapeutically plausible approach for restoring immune homeostasis (136) (**Figure 5**). Tollip (Toll-interacting protein) constitutively associates with the death domain and kinase domain of IRAK1 via its Coupling of ubiquitin conjugation to ER degradation (CUE) domain in resting cells, maintaining IRAK1 in a catalytically inhibited state and preventing premature autophosphorylation. Upon TLR/IL-1R activation, IRAK1 phosphorylates Tollip, weakening this inhibitory interaction and facilitating IRAK1 recruitment to the Myddosome (137).

The Pellino family of E3 ubiquitin ligases (Pellino-1, Pellino-2, and Pellino-3) represents a further layer of post-activation IRAK1 regulation. Pellino proteins are phosphorylated and activated by IRAK1 via their forkhead-associated (FHA) domain, which recognizes the phosphothreonine residues on IRAK1. Once activated, Pellino-1 and Pellino-2 catalyze K63-linked polyubiquitination of IRAK1, promoting downstream NF-κB and MAPK activation (138, 139). Pellino-3b, in contrast, acts as a negative regulator, stabilizing IRAK1 retention at the Myddosome and attenuating downstream signaling output. This dual isoform-dependent role of Pellino proteins in IRAK1 regulation highlights the complexity of the post-activation ubiquitination code that governs IRAK1 signaling amplitude.

SOCS1 (Suppressor of Cytokine Signaling 1) is induced as part of a negative feedback loop following TLR activation and directly associates with IRAK1 via its Src homology 2 (SH2) domain, promoting ubiquitin-mediated proteasomal degradation of IRAK1 and suppressing downstream NF-κB activation (140, 141). HS-243, a highly selective dual IRAK1/IRAK4 inhibitor developed through structural optimization of takinib, binds to the IRAK1 ATP-binding pocket with an IC50 of 24 nM and demonstrates kinome-wide selectivity across 468 kinases, with minimal residual TAK1 inhibition (IC50 0.5 μM). In primary human RA fibroblast-like synoviocytes, HS-243 suppressed LPS-induced cytokine production, pharmacologically delineating the downstream roles of IRAK1/4 and TAK1 (142). A34 is a novel ATP-competitive IRAK1 inhibitor identified through structure-based virtual screening, with an IC50 of 10.6 nM and exceptional selectivity over 215 kinases, including IRAK4. Preclinical studies have demonstrated antitumor activity in hepatocellular carcinoma (HCC) models (143). R835 (prodrug: R289; Rigel Pharmaceuticals), the first dual IRAK1/IRAK4 inhibitor to enter clinical development, demonstrated proof-of-mechanism by substantially attenuating LPS-induced serum cytokine levels in a completed phase 1 study in healthy volunteers (144). Its prodrug, R289, is currently under evaluation in a Phase Ib trial in lower-risk MDS patients refractory to prior therapies (NCT05308264), making it the most clinically advanced IRAK1-targeting agent to date (145). Compound 27 is an IRAK1/4/pan-FLT3 triple kinase inhibitor with reduced human ether-à-go-go-related gene (hERG) channel block, demonstrating preclinical activity in AML models and supporting multi-kinase targeting in FLT3-mutant and IRAK1/4-dependent leukemia models (146). UR241–2 blocks IL-1/IRAK1/4 signaling upstream of NF-κB, reducing p65/p38 phosphorylation and suppressing leukemic stem/progenitor cell (LSPC) survival in both diagnosis-stage and relapsed AML, suggesting its utility in targeting treatment-resistant leukemic stem cell compartments (147). KME-2780, a dual IRAK1/IRAK4 inhibitor (IRAK1 IC50 = 32 nM; IRAK4 IC50 = 0.9 nM), was developed to overcome compensatory IRAK1 upregulation, which limits the efficacy of IRAK4-selective inhibitors. In MDS/AML patient-derived samples, KME-2780 was significantly more effective than IRAK4-selective comparators in inducing differentiation and suppressing LSPC function, establishing co-targeting of both kinases as a superior therapeutic strategy. KME-0584 extends this approach by combining IRAK1/IRAK4 dual inhibition with pan-FLT3 activity. In preclinical AML/MDS models, KME-0584 demonstrated significant activity compared to the clinical-stage IRAK4 inhibitor CA-4948 and the FLT3 inhibitor gilteritinib across FLT3 wild-type and mutant patient samples. Favorable pharmacokinetic properties and a clean CYP450 profile support its planned clinical evaluation in relapsed/refractory AML and high-risk MDS, including hypomethylating agent (HMA) plus venetoclax-resistant patients (Kurome Therapeutics) (109, 148). Collectively, these agents reflect the evolution of the IRAK1 therapeutic landscape from single-target approaches to rational multi-kinase strategies informed by compensatory and paralog-specific signaling mechanisms.

## 9 Challenges, knowledge gaps, and future directions

### 9.1 Integrating phosphosite discovery with functional characterization

The major challenge in phosphoproteomics research is the marked disparity between the volume of phosphosite data generated through global phosphoproteomics and the functional annotation of individual phosphorylation events. The limitations of the integrated dataset are that this study analysis is subject to inherent detection biases in mass spectrometry-based phosphoproteomics, including the preferential detection of abundant, tryptic-accessible peptides and the limited representation of low-stoichiometry or highly transient phosphorylation events (149). Additionally, excluding non-class I phosphosites may have omitted functionally relevant sites with lower localization confidence levels. High-frequency IRAK1 phosphosites, such as Ser131, which were detected in 128 independent profiling datasets, represent high-priority targets for systematic functional validation. The upstream kinases, interaction-dependent functions, and disease context-specificity of these proteins remain undefined, and their functional significance cannot be inferred solely from their detection frequency. Additionally, it is necessary to develop phospho-specific antibodies for high-frequency sites that have not yet been characterized. This should be complemented by proximity ligation and co-immunoprecipitation assays conducted in physiologically relevant cell systems using experimental approaches. Phosphomimetic and phospho-null mutagenesis at recurrently detected sites, followed by the evaluation of NF-κB luciferase reporter activity, TRAF6 interaction efficiency, and ubiquitination susceptibility, can systematically explain the regulatory roles of individual phosphosites (55, 73). Additionally, mass spectrometry-compatible IRAK1 immunoprecipitation and phosphopeptide enrichment from stimulated primary immune cells are required to capture stimulus-coupled, temporally dynamic phosphorylation events that are most relevant to the biology of the disease (150, 151).

### 9.2 Context-specific signaling dynamics and temporal resolution

A fundamental limitation of static phosphoproteomic snapshots is their inability to capture the temporal dynamics of IRAK1 phosphorylation, which is critical for understanding signal encoding. Most integrated datasets represent single-timepoint analyses; time-resolved studies, where available, have been included but remain underrepresented in the current phosphoproteomic literature (152). Studies have reported that IRAK1 undergoes phosphorylation within minutes of receptor stimulation, with subsequent rapid turnover driven by proteasomal degradation. Therefore, time-resolved phosphoproteomic approaches, such as pulsed SILAC, TMT multiplexing across defined time points, and phosphoproteomics coupled with kinase inhibitor treatment, are essential for reconstructing the activation-to-degradation trajectory (153, 154). Furthermore, single-cell phosphoproteomics, although technically challenging, has the potential to resolve cell-type-specific IRAK1 phosphorylation patterns within heterogeneous tissue environments, distinguishing, for example, macrophage-specific versus epithelial IRAK1 signaling dynamics in the tumor microenvironment. The integration of phosphoproteomic data with transcriptomic and ubiquitomic datasets from matched samples will enable comprehensive systems-level modeling of IRAK1 regulatory networks.

### 9.3 Structural biology of phosphorylated IRAK1 states

Atomic-resolution structural information on phosphorylated IRAK1, particularly the hyperphosphorylated ProST domain and conformation of the activated kinase domain, remains limited. AlphaFold2 and related AI-driven structure prediction tools provide initial frameworks for modeling disordered ProST region conformations and phosphorylation-induced structural transitions (6, 111). However, experimental validation through cryo-electron microscopy (cryo-EM) of the full-length Myddosome complex in distinct activation states or through nuclear magnetic resonance (NMR) spectroscopy of phosphorylated ProST peptides is necessary to establish high-confidence structural-functional relationships. Such data would directly inform the design of phospho-state-selective inhibitors or phospho-dependent protein-protein interaction disruptors. Recent studies have expanded the repertoire of IRAK1-targeting compounds, exploring chemical scaffolds and molecular mechanisms that enhance selectivity and therapeutic efficacy, thereby broadening the potential clinical applications of IRAK1 inhibition in inflammatory and oncogenic contexts (155, 156).

### 9.4 Phospho-biomarkers and precision therapeutic targeting

The identification of disease-specific IRAK1 phosphorylation signatures could provide clinically actionable biomarkers for patient categorization and treatment monitoring. Although sites such as Ser131 and Ser607 are frequently detected across profiling datasets, there is currently a lack of systematic disease- or subtype-specific characterization, representing a clear opportunity for future studies (157). The development of validated phospho-IRAK1 immunohistochemistry assays and phospho-flow cytometry panels for primary blood cell populations would enhance the translational application of phosphoproteomic data (111, 158). Phospho-signatures could additionally assist in the selection of IRAK1 inhibitor class: patients with phosphorylation-dominant IRAK1 activation in the kinase domain may respond better to ATP-competitive inhibitors, while those with scaffold-dependent signaling may benefit from inhibitors targeting IRAK1-TRAF6 protein-protein interaction interfaces (158).

## 10 Network-level context: IRAK1 interactome and functional enrichment

To define the broader functional context of IRAK1 and prioritize network nodes for phospho-targeted intervention, IRAK1 protein interactors curated from BioGRID were analyzed, and the resulting interaction network was subjected to STRING-based biological process enrichment analysis. Our analysis revealed that the network was mainly centered on innate immune receptor signaling, with core components of the TLR and IL-1R pathways; *TLR2, TLR4, TLR9, MyD88*, Toll-interleukin 1 receptor domain-containing adaptor protein (*TIRAP)*, and *IL1R1* occupied proximal positions (159–161). Downstream signaling nodes, including *TRAF6, MAP3K7* (TAK1)*, CHUK* (IKKα)*, IKBKB* (IKKβ), and *IKBKG* (NEMO), are mapped to the canonical NF-κB and MAPK pathway modules, confirming the integrity of the IRAK1-TRAF6-TAK1-IKK signaling axis as the primary biological process enriched in this network (162, 163). Ubiquitin regulatory proteins, such as *BTRC* (β-TrCP), *CUL1, TNFAIP3* (A20), and *PELI1/2* (Pellino E3 ligases), form a prominent cluster, highlighting the pervasive role of ubiquitin-based regulation in controlling IRAK1 signal amplitude and duration (164, 165).

Transcriptional regulators, including *STAT1, STAT3, IRF4*, and *IRF7*, along with cytokine-associated proteins (IL-1β and IL-6), are prominently featured in the network periphery, indicating that IRAK1 signaling interfaces with both innate immune transcriptional programs and cytokine-driven adaptive immune regulation (166, 167). The presence of negative regulators, including *IRAK3* (IRAK-M), *SIGIRR*, and *TNIP1*, within the network provides topological evidence for built-in homeostatic feedback mechanisms that restrain IRAK1 hyperactivation (168–170). This network-level analysis positions IRAK1 as a high-centrality hub kinase, whose pharmacological inhibition is predicted to exert pleiotropic effects across multiple downstream signaling modules, consistent with the broad disease indications under clinical evaluation.

## 11 Limitations

Several limitations should be considered when interpreting the integrated IRAK1 phosphosite map. The analysis is constrained by the detection biases inherent to mass spectrometry-based phosphoproteomics, including the preferential recovery of abundant, tryptic-accessible peptides and the underrepresentation of low-stoichiometry or highly dynamic phosphorylation events. Additionally, the 246 datasets analyzed may not uniformly represent all biological contexts in which IRAK1 is active, potentially biasing the detection frequency toward over-studied cell types or stimulation conditions. Furthermore, the exclusion of non-class I phosphosites (localization probability <0.75) may have omitted functionally relevant sites with lower confidence scores. For differential datasets, published fold-change thresholds were applied when available; otherwise, ≥1.3-fold (increase) and ≤0.76-fold (decrease) with an FDR < 0.05 were used. These standardized criteria, commonly used in phosphoproteomics, facilitate comparisons across studies by minimizing technical variability. This review prioritizes the representation of phosphosite identification across independent datasets, using both profiling frequency (consistency of detection) and differential frequency (dynamic regulation) as orthogonal criteria. High-frequency sites in both contexts, such as Ser131 and Ser110, were prioritized over sites with high profiling frequency but low differential frequency (e.g., S601, T605, S371, and S556). This dual-metric approach identifies phosphosites that are both robustly detected and biologically regulated, rather than relying solely on the fold-change magnitude from individual experiments. Furthermore, the limited sequence coverage (17.1%) leaves substantial regions of IRAK1, particularly the death domain, undersampled. Most integrated datasets represent static snapshots, limiting our ability to infer the temporal dynamics of phosphorylation. Consequently, the high-frequency detection of sites such as Ser131 and Ser607 may reflect either ongoing phosphorylation or rapid turnover with a high steady-state occupancy. Distinguishing these scenarios requires time-resolved phosphoproteomics with sampling intervals of minutes to hours after TLR/IL-1R stimulation. Without temporal data, it is not possible to determine whether these sites encode activation signals or feedback regulation. Furthermore, functional annotation remains limited for most detected phosphosites, highlighting the need for targeted validation studies to establish causal relationships between phosphorylation and signaling outcomes.

## 12 Conclusions

IRAK1 acts as a multifunctional signaling regulator, and its activity is fundamentally determined by a hierarchical and spatially organized phosphorylation program. While its established role in TLR/IL-1R-NF-κB-MAPK signaling is well recognized, emerging evidence emphasizes additional non-canonical functions in nuclear transcriptional regulation, antiviral defense, and coactivation of oncogenic pathways. The organized integration of phosphoproteomic datasets in this review revealed a substantially expanded IRAK1 phosphosite landscape, comprising 18 profiling-derived and 16 differential phosphosites. Within this dataset, Ser131 and Ser110 were the most frequently detected, and several ProST-region phosphosites, such as Ser607, were recurrently observed. These detection patterns offer a data-guided framework for prioritizing sites for functional investigation; however, the detection frequency alone does not confirm functional significance. A significant gap remains between the number of identified phosphosites and the extent of their functional characterization, particularly for sites outside the kinase domain. Addressing this gap through integrative phosphoproteomic, biochemical, and structural approaches is necessary for interpreting the complete regulatory logic of IRAK1 and identifying phospho-dependent interaction interfaces suitable for specific therapeutic interventions. The availability of mechanistically distinct IRAK1 inhibitor classes, including the clinically approved pacritinib and investigational covalent inhibitor JH-X-119-01, establishes a pharmacological basis for translating mechanistic insights into precision therapeutic methods for inflammatory diseases and cancer.

Future research priorities include: (i) functional validation of high-frequency, uncharacterized phosphosites using phospho-specific antibodies and phosphomimetic mutagenesis; (ii) time-resolved, cell-type-specific phosphoproteomics to delineate the dynamic IRAK1 activation pathway in disease-relevant primary cell systems; (iii) structural characterization of phosphorylated IRAK1 states using cryo-electron microscopy and nuclear magnetic resonance; and (iv) development of phospho-IRAK1 biomarker assays for patient stratification and therapeutic monitoring. Collectively, these advances will establish IRAK1 phosphoregulation as a precision target landscape and facilitate the development of next-generation therapies for immunomodulation and cancer treatment, informed by mechanistic phosphoproteomics.
